# Multiuser Two-Dimensional Display Field Communication Using Orthogonal Frequency Subband Partitioning

**DOI:** 10.3390/s26216882

**Published:** 2026-10-30

**Authors:** Pankaj Singh, Sung-Yoon Jung

**Affiliations:** Department of Electronic Engineering, Yeungnam University, Gyeongsan 38541, Republic of Korea; pankaj_singh86@ynu.ac.kr

**Keywords:** display field communication, display-to-camera communication, optical camera communication, multiuser communication, frequency-division multiple access, two-dimensional discrete Fourier transform, spectral data-embedding, image quality

## Abstract

Display field communication (DFC) enables an electronic display to transmit hidden data to a camera while preserving the displayed visual content, but existing two-dimensional (2D) DFC studies have focused on single display–camera links. This paper proposes a simple multiuser 2D-DFC scheme in which one display conveys independent data streams to multiple camera users through nonoverlapping rectangular frequency subbands. User data are modulated using quadrature amplitude modulation, and are multiplicatively embedded into the 2D discrete Fourier transform coefficients of the input image. A reference-frame receiver with frequency-domain estimation is evaluated by using Monte Carlo simulation under equal and heterogeneous camera-noise conditions. For two users sharing a fixed total subband, the proposed scheme achieves nearly the same aggregate uncoded goodput and symbol-error performance as single-user 2D-DFC while dividing the available rate between the users. When a fixed subband is assigned to each user, the aggregate goodput increases from approximately 3 to 12 kbps as the user count increases from one to four. The corresponding mean peak signal-to-noise ratio decreases from about 50.9 to 45.8 dB, and the structural similarity index measure is higher than 0.9996. These results demonstrate a low-complexity multiple-access extension of 2D-DFC and quantify the tradeoff between throughput and visual quality.

## 1. Introduction

The extensive growth in mobile data traffic, which is projected to reach hundreds of exabytes per month globally by the end of this decade, is placing severe pressure on the radio-frequency (RF) spectrum, which is finite, heavily regulated, and increasingly congested [1,2,3]. This rising spectral scarcity has intensified interest in alternative communication paradigms that can complement existing RF systems without increasing electromagnetic interference. Visible light communication [4,5,6], which encodes data in the light source intensity operating in the optical band from 380 to 780 nm, has emerged as a promising technology. Visible light communication offers several advantages over its RF counterpart: a globally license-free optical spectrum, immunity to electromagnetic interference, natural spatial confinement that enhances security, and the ability to coexist with illumination infrastructure already deployed in homes, offices, and public spaces. With the widespread proliferation of light-emitting-diode technology and the ubiquitous presence of camera-equipped smart devices, optical camera communication [7,8], a practical incarnation of visible light communication in which an image sensor serves as the receiver, has received increased attention as an infrastructure-free, low-cost solution for short-range optical data delivery.

Among the architectures for optical camera communication, display-to-camera (D2C) communication [9] has attracted research interest. In a D2C system, an electronic display (e.g., a smartphone screen, television, laptop screen, or public digital sign) acts as the optical transmitter, and any commodity camera acts as the receiver [10]. This paradigm is compelling because it requires no dedicated hardware beyond what users already possess: billions of smartphone cameras capable of receiving and billions of electronic displays capable of transmitting. Practical application scenarios include interactive advertising and smart signage, mobile payment authentication, augmented reality overlay of contextual data, device-to-device content sharing in RF-restricted environments (e.g., hospitals, aircraft cabins, and secure facilities), and indoor optical positioning [11,12]. The global digital signage market is experiencing robust expansion with a compound annual growth rate nearing 10%, making D2C communication an economically motivated and compelling research direction [11].

Early D2C communication methods rely on visible data patterns (most prominently quick response (QR) codes [13]) that a camera can detect and decode. Although effective for low-volume, user-initiated data transfers, these approaches suffer limitations. The encoded data are visible to the human eye, interrupting or disfiguring the primary visual content of the display. The QR code occupies only a small fraction of the screen area, severely limiting spatial utilization and data throughput. Frame-by-frame code sequences are visually obtrusive, especially at lower refresh rates. These limitations prevent QR-based methods from delivering continuous, high-rate, and aesthetically unobtrusive communication over an active display. More sophisticated screen-to-camera communication schemes (e.g., InFrame [14] and InFrame++ [15]) have addressed some of these limitations by embedding data as brightness perturbations complementary to the displayed content; however, the embedding remains in the spatial domain, making it vulnerable to perspective distortion and limiting the degrees of freedom of the frequency domain.

In 2015, display field communication (DFC) [16] was proposed, which shifts the data-embedding problem to the spectral domain of the displayed image. The input image is transformed via a one-dimensional (1D) discrete Fourier transform (DFT) applied columnwise. Data symbols are multiplicatively embedded into designated mid-to-high-frequency subbands, where the human visual system (HVS) is least sensitive, and the modified image is transformed back into the spatial domain for display. At the camera receiver, division by a reference frame in the frequency domain constitutes zero-forcing (ZF) equalization, yielding the transmitted data symbols with high reliability. Because DFC applies the frequency-domain characteristics of the entire image frame, it achieves full-frame spatial parallelism while remaining visually imperceptible (even at moderate frame rates), a property related to the low-pass filtering characteristic of the HVS. The simulation results [16] demonstrated achievable data rates of about 9.5 kbps for a 256 × 256 grayscale image using 16-quadrature amplitude modulation (QAM). The authors noted the analogy between the columnwise 1D-DFT embedding of DFC and the subcarrier structure of orthogonal frequency-division multiplexing (OFDM), establishing a conceptual bridge to the extensive wireless OFDM literature.

Subsequent work has built on this conceptual foundation. In [17], the authors extended the original 1D-DFC to a 2D-DFC by adopting a full 2D-DFT for the input image, embedding data matrices in 2D corner subbands of the spectral domain, and enforcing 2D Hermitian symmetry to guarantee real-valued output. The 2D-DFC framework more accurately respects the 2D nature of image content and realizes substantially higher achievable data rates (about 125 kbps under 4-QAM) while maintaining better robustness against perspective distortion than its 1D predecessor [17]. More recent advances have focused on reducing the reference-frame overhead that halves effective throughput in 1D- and 2D-DFC. In [18], the authors proposed an iterative pilot-based scheme for 2D-DFC in which pilot symbols embedded in data frames reconstruct the reference image across successive iterations, recovering a large portion of the lost throughput. In [19], the authors introduced implicit DFC, eliminating reference frames by estimating the input image from the first-order statistics of the received frame and demonstrating an achievable data rate of up to $1.52\times 10^{5}$ bps, a 97% improvement over conventional DFC [16].

Despite this rich, rapidly expanding literature, all existing DFC work, from the original 1D-DFC [16] and 2D-DFC [17] to implicit DFC [19], has been confined to the single-user paradigm. One display transmits data to one camera, which is a significant limitation for the practical scenarios that most strongly motivate DFC. A public digital sign in a retail store, museum gallery, transit hub, or conference center is visible to multiple smartphone users, who may wish to receive personalized smart-content (e.g., targeted promotions, location-specific information, or authentication tokens) from the same screen. Similarly, in an augmented reality classroom setting, a single display may need to deliver different annotation streams concurrently to student devices. The natural extension of DFC to serve multiple users concurrently has not previously been explored.

Accurately modeling the probability of correct or incorrect detection events is a fundamental requirement for optimizing physical-layer robustness across diverse optical paradigms. This principle holds true whether evaluating macroscopic spatial-domain channels or analyzing detection vulnerabilities in highly sensitive systems such as quantum key distribution networks [20]. Thus, existing DFC frameworks have yet to extend these analytical principles to a multiuser (MU) environment. To solve this problem, a multiple-access DFC architecture must physically isolate independent data streams to prevent interuser interference (IUI) while maintaining predictable error probabilities for each link. In RF wireless communications, the MU problem is well understood. Orthogonal frequency-division multiple access (OFDMA) [21] allocates disjoint sets of subcarriers to users, achieving interference-free multiple access at the cost of per-user bandwidth reduction. In 2D-DFC [17], the frequency domain of a P×Q image is partitioned into subbands indexed by row and column frequency. To enable multiple users, the 2D frequency subband can be partitioned among *K* concurrent DFC users. Each user is assigned a nonoverlapping set of row-indexed frequency bins, and their data are independently embedded using Hadamard multiplication. The camera for each user independently extracts only its assigned subband after ZF equalization, with zero IUI by construction. This conceptual mapping motivates a clean and tractable MU extension of 2D-DFC.

This paper proposes and analyzes MU-2D-DFC, the first multiple-access DFC scheme in which a single display simultaneously serves *K* camera users through orthogonal frequency subband partitioning. The contributions of this paper are as follows.

1. A formal mathematical framework for MU-2D-DFC is derived by extending 2D-DFC [17] to *K* users with rowwise subband assignment.
2. This work analyzes the case in which all *K* users experience the same signal-to-noise ratio (SNR). The simulation results demonstrate that the per-user symbol-error rate (SER) and goodput scale predictably with the number of users. Moreover, when the available subband is partitioned among the users, the total sum rate equals the single-user 2D-DFC rate.
3. This work also analyzes the practically relevant case in which users are at various distances or viewing angles with respect to the display and experience different effective SNRs.
4. This study demonstrates that when each user retains a fixed per-user subband allocation and additional users are admitted by expanding the total occupied subband, the system sum rate increases nearly proportionally to *K*. This outcome represents a direct MU multiplexing gain, achieved at the cost of gradual and quantifiable degradation in the visual quality of the displayed image, measured in terms of the peak SNR (PSNR).
5. Finally, the tradeoff between the visual quality of the displayed image and the number of concurrent users is characterized analytically and validated via simulation. The results reveal that the PSNR is well above the commonly adopted 30dB perceptual-quality threshold, even when K=4 users are served concurrently.

The remainder of this paper is organized as follows. Section 2 presents the MU-2D-DFC system model, including the subband partitioning and data-embedding procedure. Next, Section 3 describes the D2C channel model in this study. Then, Section 4 presents the ZF decoding framework and goodput computation, and Section 5 presents the empirical AWGN performance. Section 6 analyzes practical deployment considerations, including spatial clipping, geometric distortion, and input-image dependency. Finally, Section 7 concludes the paper.

## 2. Multiuser Two-Dimensional Display Field Communication System Model

### 2.1. System Overview

**Figure 1:**
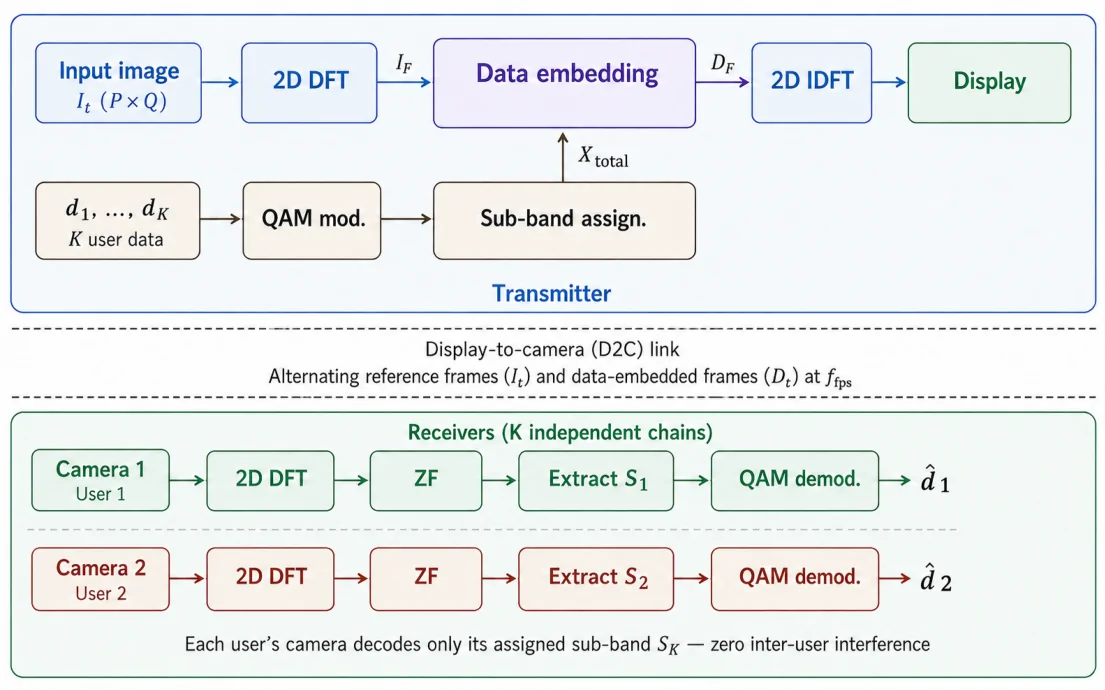
System model of the proposed multiuser two-dimensional display field communication (MU-2D-DFC) scheme (for K=2 users).

Figure 1 illustrates the proposed MU-2D-DFC system. A single P×Q grayscale electronic display simultaneously transmits independent data streams to *K* camera-equipped users. The display acts as a broadcast transmitter, embedding the data for each user into a dedicated, nonoverlapping region of the 2D frequency domain of the input image. Because the embedding is multiplicative and confined to mid-to-high-frequency subbands where the HVS is least sensitive [16], the displayed content appears visually identical to the unmodified input image. At the receiver side, each user camera independently captures the displayed frame sequence, applies a 2D DFT, and extracts the transmitted data from its own assigned subband using ZF equalization. Because the subbands assigned to different users are orthogonal by construction, no IUI arises, and the *K* users are served simultaneously without coordination between receivers.

**Figure 2:**
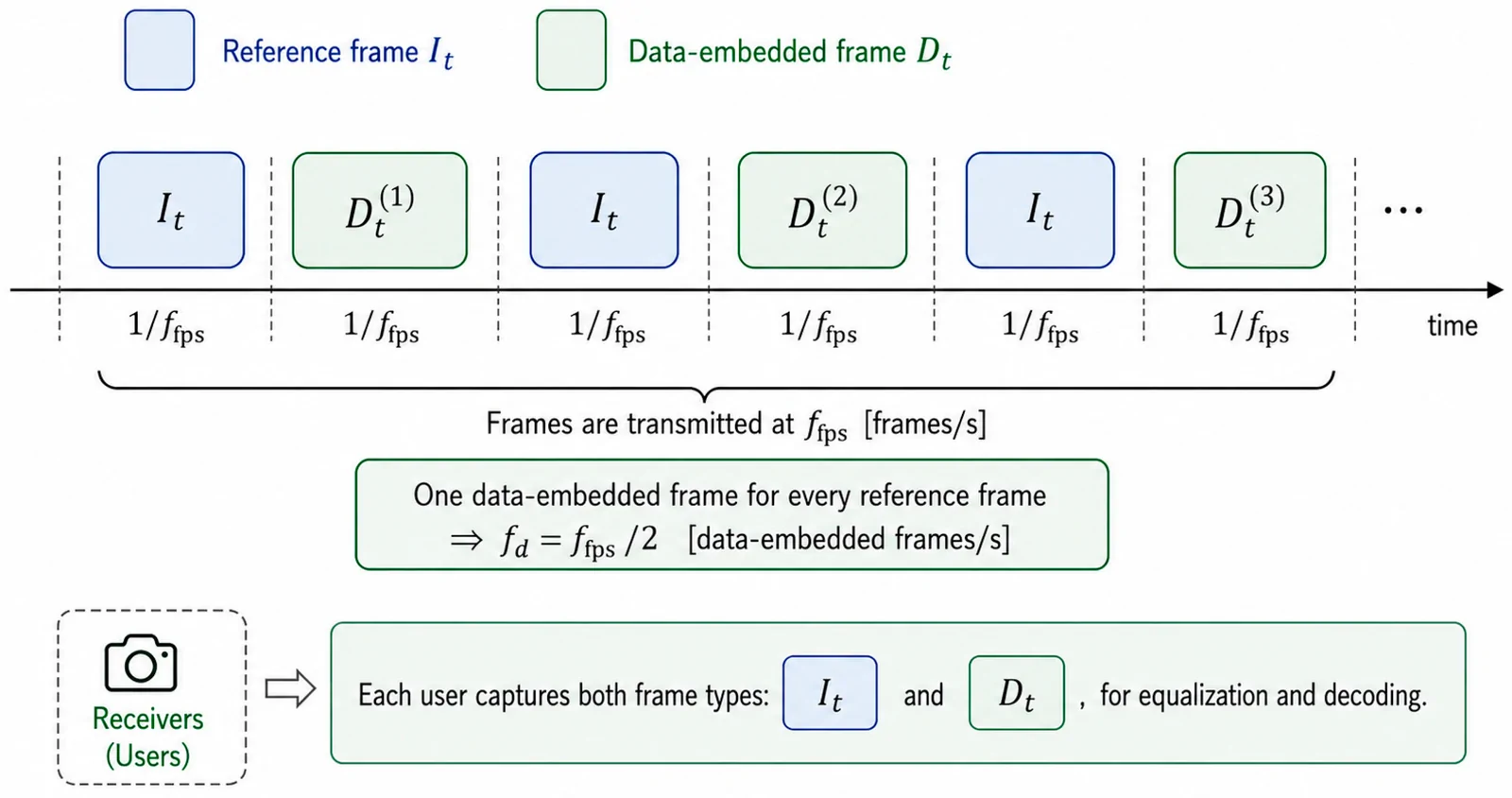
Alternating reference/data frame sequence over the display-to-camera (D2C) link. One data-embedded frame is transmitted per reference frame, yielding $f_{d}=f_{\mathrm{fps}}/2$.

As depicted in Figure 2, the frame sequence alternates between reference frames (unmodified input image) and data-embedded frames [16,17]. Reference frames enable the receiver to estimate and invert the D2C channel without requiring channel feedback. One data-embedded frame is transmitted for every reference frame, yielding an effective data frame rate of

(1) $$f_{d}=\frac{f_{\mathrm{fps}}}{2},$$

in frames per second, where $f_{\mathrm{fps}}$ denotes the display refresh rate.

### 2.2. Transformation of the Input Image

Let $I_{t}\in R^{P\times Q}$ denote the input image in the spatial domain. The 2D DFT of the input image is computed as [17]

(2) $$I_{F}=F_{P}\;I_{t}\;F_{Q},$$

where $F_{P}\in C^{P\times P}$ and $F_{Q}\in C^{Q\times Q}$ are the DFT matrices of dimensions *P* and *Q*, respectively, with the (m,n)th elements ${\left[F_{P}\right]}_{m,n}=e^{-j\frac{2\pi }{P}\left(m-1\right)\left(n-1\right)}$ and ${\left[F_{Q}\right]}_{m,n}=e^{-j\frac{2\pi }{Q}\left(m-1\right)\left(n-1\right)}$. In the 2D DFT representation (without a frequency shift), low-frequency components are at the four corners of the P×Q frequency-domain array, whereas the highest-frequency components are concentrated near the center [17]. The DC component is at index (1,1) (using 1-based indexing throughout this work), and the frequency increases monotonically toward the index (⌊P/2⌋+1,⌊Q/2⌋+1). Afterward, the component decreases symmetrically due to conjugate periodicity. Because $I_{t}$ is real-valued, its 2D DFT satisfies the conjugate (Hermitian) symmetry property calculated as follows:

(3) $$I_{F}\left(p,q\right)=I_{F}^{*}\left(P-p+2,\;Q-q+2\right),$$

for 2≤p≤P and 2≤q≤Q. The first row (p=1), first column (q=1), and DC component are self-conjugate under the general modulo form of the symmetry, $I_{F}\left(p,q\right)=I_{F}^{*}\;\left(\left((P-p+1)\;\mathrm{mod}\;P\right)+1,\;\left((Q-q+1)\;\mathrm{mod}\;Q\right)+1\right)$, are real-valued or pairwise conjugate within themselves, and are not used for data embedding in this study. This symmetry must be preserved in the data-embedded frequency-domain image to guarantee that the inverse 2D DFT produces a real-valued spatial-domain image suitable for display.

### 2.3. Subband Partitioning

**Figure 3:**
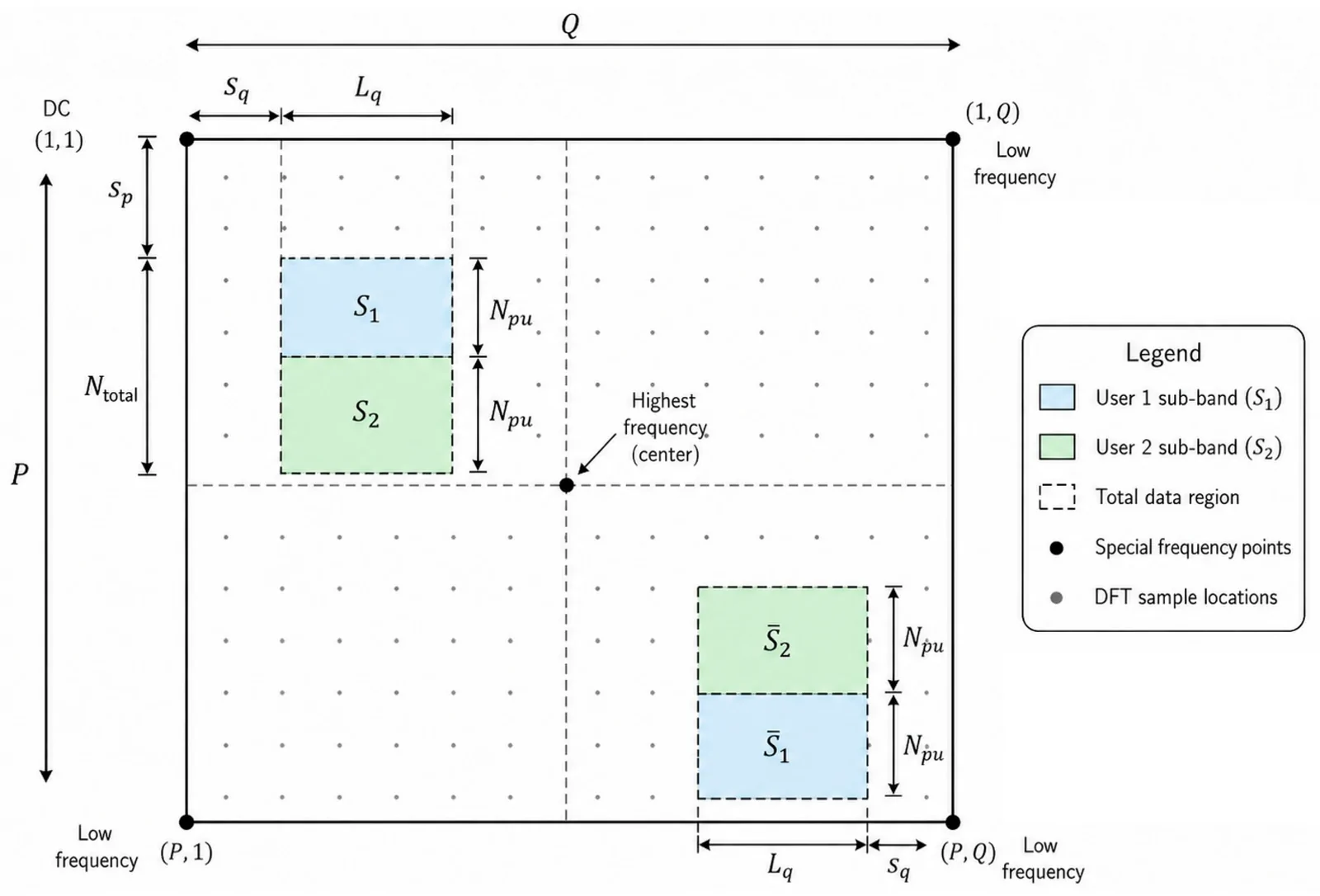
Orthogonal subband partitioning for K=2 users.

In Figure 3, the total available data region in the top-left quadrant of the 2D DFT domain is a rectangular block occupying rows $\left[s_{p},\;s_{p}+N_{\mathrm{total}}-1\right]$ and columns $\left[s_{q},\;s_{q}+L_{q}-1\right]$, where $s_{p}\left(\geq 2\right)$ and $s_{q}\left(\geq 2\right)$ represent the subband start indices, $N_{\mathrm{total}}$ denotes the total number of data rows, and $L_{q}$ indicates the number of data columns. These parameters satisfy the Hermitian symmetry bounds [17]:

(4) $$s_{p}+N_{\mathrm{total}}<\frac{P}{2}+2$$

and

(5) $$s_{q}+L_{q}<\frac{Q}{2}+2,$$

which ensure the data region and its conjugate mirror do not overlap in either dimension.

In MU-2D-DFC, $N_{\mathrm{total}}$ available rows are partitioned equally among *K* users. Each user *k* (k=1,2,…,K) is assigned $N_{p_{u}}=\left\lfloor N_{\mathrm{total}}/K\right\rfloor$ consecutive rows. Therefore, the row range for user *k* is

(6) $$r_{s}^{\left(k\right)}=s_{p}+\left(k-1\right)N_{p_{u}},\;r_{e}^{\left(k\right)}=s_{p}+k\cdot N_{p_{u}}-1,$$

where $r_{s}^{\left(k\right)}$ and $r_{e}^{\left(k\right)}$ represent the starting and ending row indices, respectively, of the *k*th user. The column range is the same for all users, as

(7) $$c_{s}=s_{q},\;c_{e}=s_{q}+L_{q}-1,$$

where $c_{s}$ and $c_{e}$ denote the starting and ending indices, respectively, for the columns. Therefore, the subband set assigned to user *k* is defined as follows:

(8) $$S_{k}=\left\{\left(p,q\right)\;|\;r_{s}^{\left(k\right)}\leq p\leq r_{e}^{\left(k\right)},\;c_{s}\leq q\leq c_{e}\right\},$$

with $|S_{k}|=N_{p_{u}}\times L_{q}$ frequency-domain elements per user.

For the successful implementation of the proposed MU-2D-DFC scheme, the proposed subband partitioning must satisfy two properties.

- Orthogonality: The subbands must be nonoverlapping by construction. Mathematically,
  (9) $$S_{i}\cap S_{j}=\emptyset \;\mathrm{for}\;\mathrm{all}\;i\neq j,$$
  because the row ranges in (6) are disjoint for distinct users. This orthogonality is the fundamental property that guarantees zero IUI at the receiver without coordination among users.
- Hermitian symmetry [16,17]: The conjugate mirror of $S_{k}$ is defined as follows:
  (10) $${\bar{S}}_{k}=\left\{\left(P-p+2,\;Q-q+2\right)\;|\;\left(p,q\right)\in S_{k}\right\},$$
  which occupies rows $\left[P-r_{e}^{\left(k\right)}+2,\;P-r_{s}^{\left(k\right)}+2\right]$ and columns $\left[Q-c_{e}+2,\;Q-c_{s}+2\right]$ in the bottom-right quadrant (cf. Figure 3). The conditions (4) and (5) guarantee that $S_{k}\cap {\bar{S}}_{k}=\emptyset$ and that $S_{i}\cap {\bar{S}}_{j}=\emptyset$ for all i,j, ensuring that neither the data regions nor their mirrors overlap one another.

### 2.4. Per-User Data Symbols and Global Data Matrix

Let $d_{k}\in A^{N_{p_{u}}\times L_{q}}$ denote the QAM symbol matrix for user *k*, where A denotes an *M*-ary QAM constellation. The symbols in $d_{k}$ are normalized to the unit average power as follows:

(11) $$E\left[{\left|d_{k}\left(r,c\right)\right|}^{2}\right]=1\;\mathrm{for}\;\mathrm{all}\;\left(r,c\right),$$

where E[·] represents the expectation operator. Hence, any difference in the received reliability among users is caused by the assigned frequency coefficients and channel noise, rather than by different symbol powers. The global P×Q data matrix $X_{\mathrm{total}}$ is constructed as follows:

(12) $$X_{\mathrm{total}}\left(p,q\right)=\left\{\begin{array}{cc} d_{k}\;\left(p-r_{s}^{\left(k\right)}+1,\;q-c_{s}+1\right),\; & \mathrm{if}\;\left(p,q\right)\in S_{k}\;\mathrm{for}\;\mathrm{some}\;k,\\ d_{k}^{*}\;\left(P-p-r_{s}^{\left(k\right)}+3,\;Q-q-c_{s}+3\right),\; & \mathrm{if}\;\left(p,q\right)\in {\bar{S}}_{k}\;\mathrm{for}\;\mathrm{some}\;k,\\ 1,\; & \mathrm{otherwise}. \end{array}\right.$$

The first case places the QAM symbols for user *k* in the designated top-left subband. The second case places the conjugate-reflected symbols in the corresponding bottom-right Hermitian mirror, which is equivalent to

(13) $${\left.X_{\mathrm{total}}\right|}_{{\bar{S}}_{k}}={\mathrm{rot}}_{180}\;\left(d_{k}^{*}\right),$$

where ${\mathrm{rot}}_{180}\left(\cdot \right)$ denotes the simultaneous reversal of rows and columns. The value 1 assigned to all nonsubband elements in (12) ensures that multiplying by $X_{\mathrm{total}}$ leaves the input-image frequency coefficients that are outside the data subbands unchanged. Together, the first and second cases of (12) enforce (3) on $X_{\mathrm{total}}$ elementwise, which is a necessary and sufficient condition for the embedded spatial-domain image to be real-valued after 2D inverse DFT.

### 2.5. Data Embedding

The data-embedded frequency-domain image is formed using the Hadamard product as follows:

(14) $$D_{F}=I_{F}⊙X_{\mathrm{total}}.$$

In MU-2D-DFC, the 2D frequency coefficients of the input image serve as the equivalent of OFDM subcarriers [21]. The data symbol $d_{k}\left(r,c\right)$ of user *k* scales the image coefficient $I_{F}\left(r_{s}^{\left(k\right)}+r-1,\;c_{s}+c-1\right)$ at the corresponding position in $S_{k}$. At positions outside the subbands, $X_{\mathrm{total}}\left(p,q\right)=1$, so $D_{F}\left(p,q\right)=I_{F}\left(p,q\right)$, and the input image is unmodified. The perturbation to the input-image frequency coefficients is confined to the union of all data subbands and their Hermitian-symmetric counterparts as follows:

(15) $$D_{F}-I_{F}=I_{F}⊙\left(X_{\mathrm{total}}-1\right),$$

where 1 denotes the matrix of all 1. To display the image on the screen, the data-embedded image must be converted back to the spatial domain. This conversion is obtained using the 2D inverse DFT operation:

(16) Dt=Re1PQFPHDFFQH,

where ${\left(\cdot \right)}^{H}$ denotes the conjugate transpose and Re(·) denotes the real-part operator, which is applied as a numerical safeguard. Theoretically, $D_{t}$ is real-valued by the construction of $X_{\mathrm{total}}$ in (12). Finally, the resulting image $D_{t}$ is quantized to 8-bit unsigned integers (pixel values in [0,255]) and displayed on the electronic screen.

A fundamental requirement of displaying the data-embedded frame is converting the real-valued spatial-domain matrix back into 8-bit unsigned integers, which necessitates clipping the pixel values to a strict [0, 255] range. As with PAPR clipping in RF OFDM systems, this is a nonlinear operation that theoretically generates out-of-band spectral leakage, threatening subband orthogonality and introducing IUI. However, because all simulated MU-2D-DFC configurations achieve an observed PSNR ≥45.8 dB, the embedding amplitude perturbation remains exceptionally small, as quantified in Section 6.

## 3. Display-to-Camera Channel Model

Each user *k* captures two types of image frames with the camera: reference and data-embedded frames. The D2C channel introduces additive noise due to photon shot noise, ambient-light interference, camera sensor noise, and quantization effects. Following the established DFC channel model [16,17], the received reference and data frames for the camera of user *k* are modeled as

(17) $$Z_{t}^{\left(k\right)}=I_{t}+N_{t}^{\left(k\right)}$$

(18) $$Y_{t}^{\left(k\right)}=D_{t}+N_{t}^{\prime (k)},$$

where $N_{t}^{\left(k\right)}$ and $N_{t}^{\prime (k)}$ are mutually independent AWGN matrices with zero mean and per-element variance $\sigma_{k}^{2}$, which may differ among users when modeling heterogeneous-channel conditions (e.g., diverse viewing distances or angles). Additionally, $I_{t}$ and $D_{t}$ are the same for all *K* users (because they are viewing the same display), whereas the noise realizations are independent for each camera.

In physical DFC implementations, perspective distortion caused by off-axis camera geometry is typically compensated for prior to data extraction using standard preprocessing techniques (e.g., Harris corner detection and Hough transform boundary alignment) [22]. To mathematically isolate the fundamental multiple-access performance and theoretical capacity limits of the proposed orthogonal subband partitioning, these geometric preprocessing blocks are deliberately bypassed in our current Monte Carlo evaluations. Consequently, the idealized AWGN-based channel model utilized in this study is practically valid only under strictly controlled, near-laboratory conditions: perfect frontal or mathematically restored spatial registration, a static display-camera geometric relationship, accurate optical focus, and a perfectly linear radiometric response. When these conditions are not met, uncompensated physical nonidealities—such as nonuniform display brightness, image resampling errors, and nonlinear camera responses (e.g., gamma)—introduce spatial mismatches and inter-modulation products that severely distort the ZF equalization process (22) and destroy the strict subband orthogonality required for zero IUI. To fully realize the proposed MU-2D-DFC architecture in complex, fluctuating real-world environments, future implementations must integrate adaptive optical communication receivers [23] and techniques for resilient optical information transmission [24]. A comprehensive analysis of how these specific geometric, photometric, and temporal impairments degrade the proposed architecture is provided in Section 6. Applying the 2D DFT to both sides of (17) and (18) results in

(19) $$Z_{F}^{\left(k\right)}=F_{P}\;Z_{t}^{\left(k\right)}\;F_{Q}=I_{F}+N_{F}^{\left(k\right)}$$

(20) $$Y_{F}^{\left(k\right)}=F_{P}\;Y_{t}^{\left(k\right)}\;F_{Q}=D_{F}+N_{F}^{\prime (k)},$$

where $N_{F}^{\left(k\right)}=F_{P}N_{t}^{\left(k\right)}F_{Q}$ and $N_{F}^{\prime (k)}=F_{P}N_{t}^{\prime (k)}F_{Q}$ denote the 2D DFT representations of the noise matrices. Substituting (14) into (20) yields

(21) $$Y_{F}^{\left(k\right)}=I_{F}⊙X_{\mathrm{total}}+N_{F}^{\prime (k)}.$$

## 4. Receiver Processing

### 4.1. Zero-Forcing Equalization

At the camera of user *k*, ZF equalization is performed using the elementwise division of the received data-frame DFT by the received reference-frame DFT as follows:

(22) $${\hat{X}}^{\left(k\right)}=Y_{F}^{\left(k\right)}⊘Z_{F}^{\left(k\right)},$$

where ⊘ denotes elementwise division. After substituting (19) to (21) into (22), the estimated data value at position (p,q) is calculated as

(23) $${\hat{X}}^{\left(k\right)}\left(p,q\right)=\frac{I_{F}\left(p,q\right)\cdot X_{\mathrm{total}}\left(p,q\right)+N_{F}^{\prime (k)}\left(p,q\right)}{I_{F}\left(p,q\right)+N_{F}^{\left(k\right)}\left(p,q\right)}.$$

For $\left(p,q\right)\in S_{k}$, $X_{\mathrm{total}}\left(p,q\right)=d_{k}\left(p-r_{s}^{\left(k\right)}+1,\;q-c_{s}+1\right)$. In the high-SNR regime (i.e., $\left|I_{F}\left(p,q\right)\right|\gg \left|N_{F}^{\left(k\right)}\left(p,q\right)\right|$), a first-order Taylor expansion of (23) results in the following approximate expression (Appendix A):

(24) $${\hat{X}}^{\left(k\right)}\left(p,q\right)\approx d_{k}\left(r,c\right)+\frac{N_{F}^{\prime (k)}\left(p,q\right)-d_{k}\left(r,c\right)\cdot N_{F}^{\left(k\right)}\left(p,q\right)}{I_{F}\left(p,q\right)}.$$

The second term in (24) constitutes the residual noise after equalization. Each element of $N_{F}^{\left(k\right)}=F_{P}N_{t}^{\left(k\right)}F_{Q}$ is a linear combination of PQ independent spatial-domain noise samples with unit-magnitude weights; thus, the per-coefficient frequency-domain noise variance is $E\;[|N_{F}^{\left(k\right)}\left(p,q\right){|}^{2}]=PQ\;\sigma_{k}^{2}$. Therefore, the variance at position (p,q) is

(25) $$\sigma_{\mathrm{eff}}^{2}\left(p,q\right)\approx \frac{PQ\;\sigma_{k}^{2}\left(1+E\;\left[{\left|d_{k}\right|}^{2}\right]\right)}{{\left|I_{F}\left(p,q\right)\right|}^{2}}=\frac{2PQ\;\sigma_{k}^{2}}{{\left|I_{F}\left(p,q\right)\right|}^{2}},$$

where the last equality uses the unit-power normalization in (11). The effective per-coefficient SNR experienced by user *k* at position (p,q) is (Appendix B)

(26) $$\gamma_{k}\left(p,q\right)=\frac{E\;\left[{\left|d_{k}\right|}^{2}\right]}{\sigma_{\mathrm{eff}}^{2}\left(p,q\right)}=\frac{{\left|I_{F}\left(p,q\right)\right|}^{2}}{2PQ\;\sigma_{k}^{2}}.$$

Three principal observations follow from (26):

- Image-content dependence: The effective SNR is proportional to ${\left|I_{F}\left(p,q\right)\right|}^{2}$, the squared magnitude of the input-image frequency coefficient at position (p,q). Because this magnitude typically varies across the 2D frequency plane, users (assigned to different row bands) experience systematically different effective SNR even under identical noise conditions $\left(\sigma_{k}^{2}=\sigma^{2}\right)$.
- Independent decoding: For $\left(p,q\right)\in S_{k}$, (23) depends on only $d_{k}$ and no other user symbols ${\left\{d_{j}\right\}}_{j\neq k}$, confirming the zero-IUI property stated in (9). User *k* can decode data using only the user’s own subband coefficients, without knowledge of or interference from other K−1 users.
- Noise amplification at weak coefficients: Positions (p,q) where $\left|I_{F}\left(p,q\right)\right|$ is small produce large effective noise values, as observed in (25). This result motivates selecting subbands in frequency regions where the input image has substantial energy, typically in the mid-frequency range.

User *k* recovers data exclusively from ${\left.{\hat{X}}^{\left(k\right)}\right|}_{S_{k}}$, which is the restriction of (22) to the rows and columns of $S_{k}$. Finally, hard-decision QAM demodulation is applied to each element of this submatrix to obtain the estimated symbol matrix ${\hat{d}}_{k}$.

### 4.2. Goodput

The goodput for user *k* is defined as the rate of correctly received uncoded bits [16,17], as

(27) $$R_{k}=f_{d}\cdot N_{p_{u}}\cdot L_{q}\cdot \log_{2}M\cdot \left(1-{\mathrm{BER}}_{k}\right),$$

where $f_{d}=f_{\mathrm{fps}}/2$ denotes the effective data frame rate (in fps), $N_{p_{u}}\cdot L_{q}$ represents the number of QAM symbols per data frame for user *k*, $\log_{2}M$ indicates the number of bits per symbol, and ${\mathrm{BER}}_{k}$ denotes the bit error rate of user *k*. Note that while the system’s fundamental reliability is visualized using the SER, the uncoded goodput is strictly calculated using the BER to account for correctly received bits within erroneous symbols under Gray mapping. Thus, the total system sum rate is

(28) $$R_{\mathrm{sum}}=\sum_{k=1}^{K}R_{k}=f_{d}\cdot K\cdot N_{p_{u}}\cdot L_{q}\cdot \log_{2}M\cdot \left(1-\frac{1}{K}\sum_{k=1}^{K}{\mathrm{BER}}_{k}\right).$$

For the equal-channel scenario (i.e., ${\mathrm{BER}}_{k}=\mathrm{BER}$ for all *k*, with fixed total bandwidth $K\cdot N_{p_{u}}=N_{\mathrm{total}}$), expression (28) simplifies to

(29) $$R_{\mathrm{sum}}=f_{d}\cdot N_{\mathrm{total}}\cdot L_{q}\cdot \log_{2}M\cdot \left(1-\mathrm{BER}\right),$$

which is identical to the goodput of a single-user 2D-DFC system occupying the full $N_{\mathrm{total}}$-row subband [17]. This result confirms that subband partitioning among *K* users does not sacrifice total system throughput in the equal-channel case. The multiple-access capability arises from serving multiple users concurrently at reduced per-user rates.

When each user is guaranteed a fixed minimum subband allocation of $N_{p_{u}}$ rows (i.e., the total subband expands with *K*), the sum rate becomes

(30) $$R_{\mathrm{sum}}=f_{d}\cdot K\cdot N_{p_{u}}\cdot L_{q}\cdot \log_{2}M\cdot \left(1-\mathrm{BER}\right),$$

which increases linearly with *K*, provided the Hermitian symmetry constraints of (4) and (5) are not violated. This linear scaling constitutes the multiplexing gain of MU-2D-DFC and is analyzed in the next section.

## 5. Simulation Results

This section presents the simulation results of the proposed MU-2D-DFC scheme. First, Section 5.1 verifies the visual unobtrusiveness of the MU embedding. Then, Section 5.2 and Section 5.3 analyze the communication performance under the equal- and heterogeneous-channel scenarios, respectively. Next, Section 5.4 examines the per-user goodput and bandwidth-conservation property of subband partitioning. Finally, Section 5.5 presents the results for the tradeoff between the multiplexing gain and PSNR.

Table 1 presents the default simulation parameters. Moreover, throughout the simulation results, the horizontal axis represents the global DFC embedding SNR, denoted as $\gamma_{\mathrm{global}}$. This metric is defined as the ratio of the mean-squared spatial-domain embedding perturbation to the differential camera-noise power:

(31) $$\gamma_{\mathrm{global}}=10\log_{10}\left(\frac{\frac{1}{PQ}E\left[∥D_{t}-I_{t}{∥}_{F}^{2}\right]}{2\sigma_{k}^{2}}\right).$$

By defining the axis in terms of the spatial-domain noise variance $\sigma_{k}^{2}$, the simulation sweep is connected to the analytical model in (26). As $\gamma_{\mathrm{global}}$ increases, $\sigma_{k}^{2}$ decreases, which proportionately increases the localized, per-coefficient effective SNR $\gamma_{k}\left(p,q\right)$ for all users.

All numerical evaluations were implemented and executed in MATLAB R2026b. To ensure statistical reliability and fully capture the residual error behavior, each data point on the SER and goodput curves was averaged over $10^{5}$ independent Monte Carlo trials. For strict reproducibility, the pseudorandom number generator was initialized using a fixed seed (Mersenne Twister, seed = 42) prior to each simulation loop. During each trial, independent data frames were generated using random QAM symbols. The AWGN for the reference and data-embedded frames ($N_{t}^{\left(k\right)}$ and $N_{t}^{\prime (k)}$) was generated using independent, zero-mean normal distributions scaled to the respective variance $\sigma_{k}^{2}$ corresponding to the target global DFC embedding SNR for that iteration.

In addition to the SER and goodput defined in the previous section, a DFC system must not distort the original image too much so that the display keeps serving its original purpose. Therefore, the visual quality of the data-embedded image must be closer to the original image. This visual quality of the data-embedded image relative to the original image is quantified by PSNR, which is defined as

(32) $$\mathrm{PSNR}=10\log_{10}\;\left(\frac{255^{2}}{\frac{1}{PQ}\;{∥D_{t}-I_{t}∥}_{F}^{2}}\right)\;\left[\mathrm{dB}\right],$$

where ${∥\cdot ∥}_{F}$ denotes the Frobenius norm. Notably, a PSNR higher than 30 dB is regarded as the threshold for visually imperceptible distortion [16,17]. In addition, this work evaluates the SSIM [25,26], a perceptual metric for how similar two images are, designed to match human visual judgment better than a pixelwise metric (e.g., PSNR).

Although PSNR and SSIM serve as standard, computationally efficient benchmarks for DFC visual evaluation, they may not perfectly capture complex HVS characteristics under diverse display settings. Recent developments in learning-based image-quality assessment [27] provide highly intelligent frameworks for more accurately evaluating perceptual distortion. Integrating such learning-based metrics could enable more subtle, human-centric visual quality optimization for future MU-2D-DFC systems.

### 5.1. Visual Quality of the MU Embedded Image

**Figure 4:**
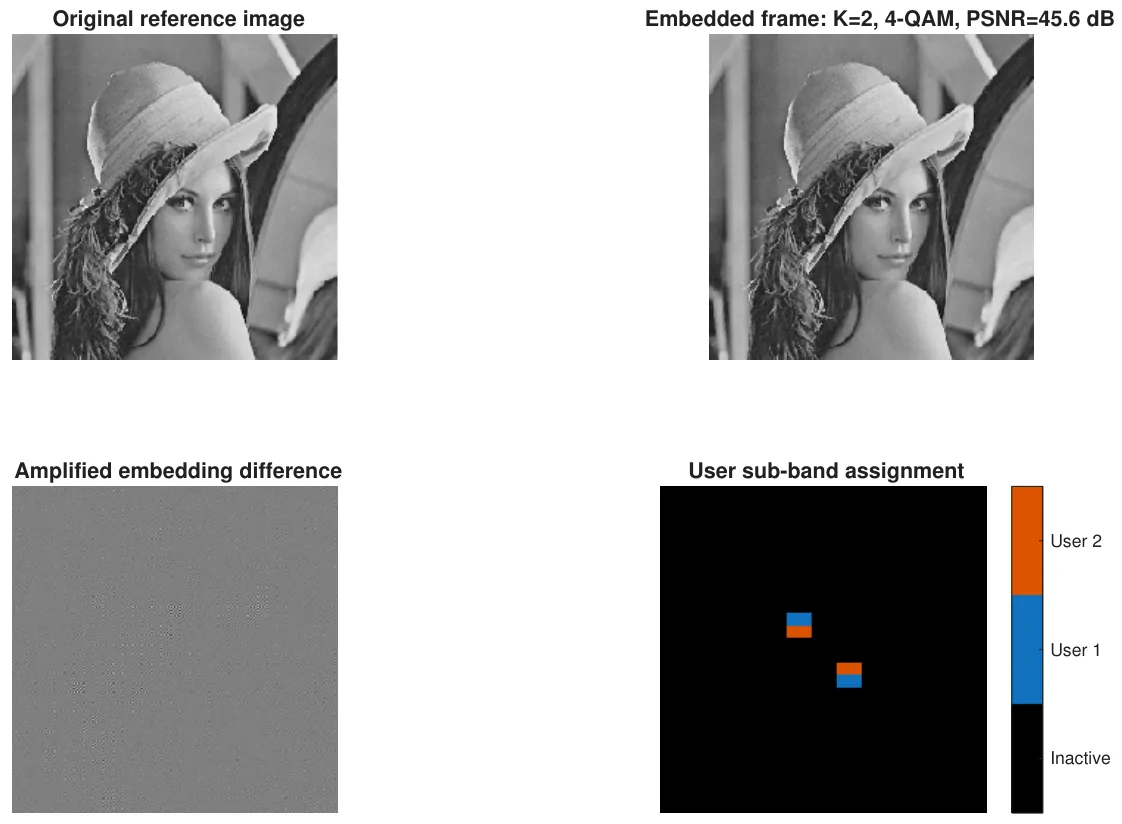
Original and multiuser data-embedded frames with the corresponding subband assignment map (K=2, 4-quadrature amplitude modulation (QAM), PSNR=45.6dB).

The visual effect of the proposed MU-2D-DFC data embedding is evaluated. Figure 4 depicts the original reference frame, data-embedded frame, amplified embedding difference, and user subband assignment map. The top two panels compare the original reference frame and data-embedded frame. The data-embedded frame attains a PSNR of 45.6dB, far higher than the 30dB perceptual threshold, indicating that the embedded data are visually unobtrusive. The amplified-difference image (bottom-left panel) confirms that the perturbation is a low-amplitude, spatially spread texture rather than a localized artifact, a direct consequence of embedding in the DFT domain, where each modified coefficient contributes a global, low-energy sinusoidal pattern in the spatial domain. Finally, the subband assignment map (bottom-right panel) visualizes the orthogonal partition. The row bands (blue/orange) of the two users occupy the mid-frequency region along with their point-symmetric Hermitian mirrors, and all remaining coefficients (black) are inactive (i.e., carrying an unmodified input image). The map confirms that the two users are allocated nonoverlapping frequency-domain regions. The PSNR of 45.6 dB for this specific K=2 configuration, which utilizes an expansive total allocation of $N_{total}=20$ rows, naturally differs from the K=2 data point plotted later in Section 5.5.2, which utilizes a much narrower fixed bandwidth of $N_{total}=10$ rows.

### 5.2. Scenario A: Equal-Channel Condition

**Figure 5:**
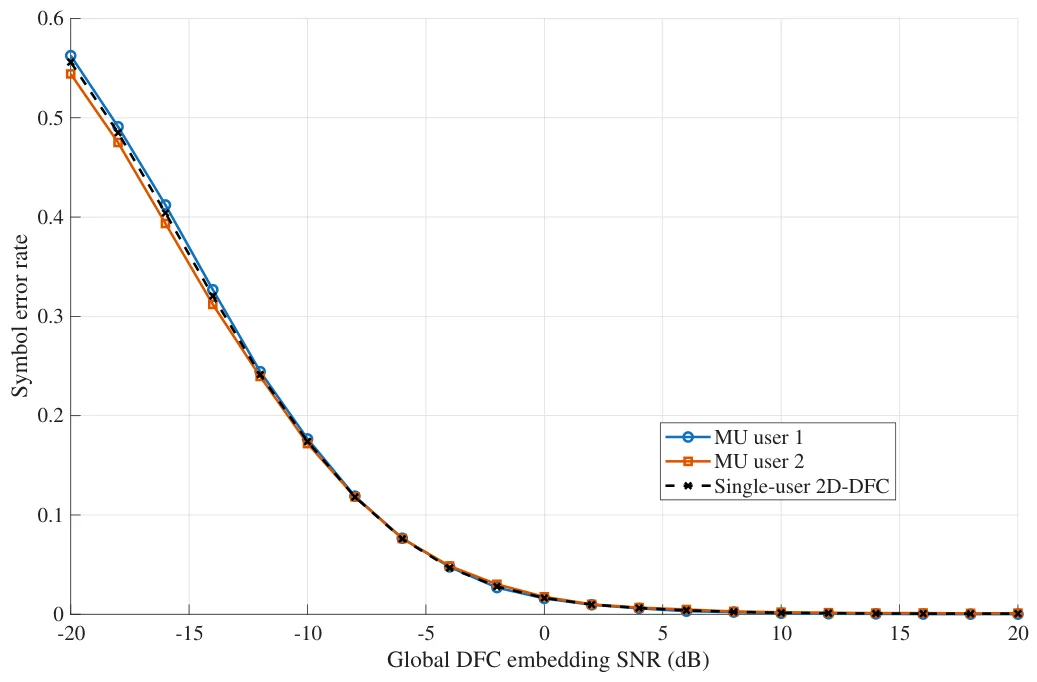
Symbol error rate (SER) performance of the multiuser two-dimensional display field communication (MU-2D-DFC) under the equal-channel scenario.

Figure 5 presents the SER performance of the proposed MU-2D-DFC scheme under the equal-channel scenario, where both users experience the same camera-noise variance.

First, the SER curves for Users 1 and 2 are practically coincident over the entire SNR range, confirming that the rowwise orthogonal subband partition provides statistically similar and interference-free data recovery for both users. The small residual difference between the two users is due to the image-content-dependent effective SNR because the two users occupy slightly different frequency rows of the input-image spectrum. Because MU-2D-DFC is the first multiple-access scheme proposed for D2C links, no existing MU DFC frameworks serve as direct comparative baselines. Therefore, the state-of-the-art single-user 2D-DFC system [17] is employed as the baseline for comparison. In Figure 5, the single-user SER curve closely follows the two MU-2D-DFC user curves, demonstrating that dividing the total subband between two users does not introduce an additional SER penalty. This result confirms that the proposed multiple-access mechanism is orthogonal by construction, where each receiver extracts only its own assigned subband, and no IUI appears in the ZF-equalized symbols.

Additionally, as the SNR increases, the SER decreases monotonically. At low SNR values, the SER—which represents the empirical probability of the incorrect-detection event, $P(\hat{s}\neq s)$—is high because both the reference- and data-frame DFT coefficients are strongly affected by noise, as governed by the effective SNR in (26). As the SNR increases, the ZF equalizer becomes increasingly reliable, and the SER approaches zero beyond about 5dB. This result validates the analytical prediction that MU-2D-DFC preserves single-user reliability while enabling simultaneous transmission to multiple camera users.

### 5.3. Scenario B: Heterogeneous-Channel Condition

**Figure 6:**
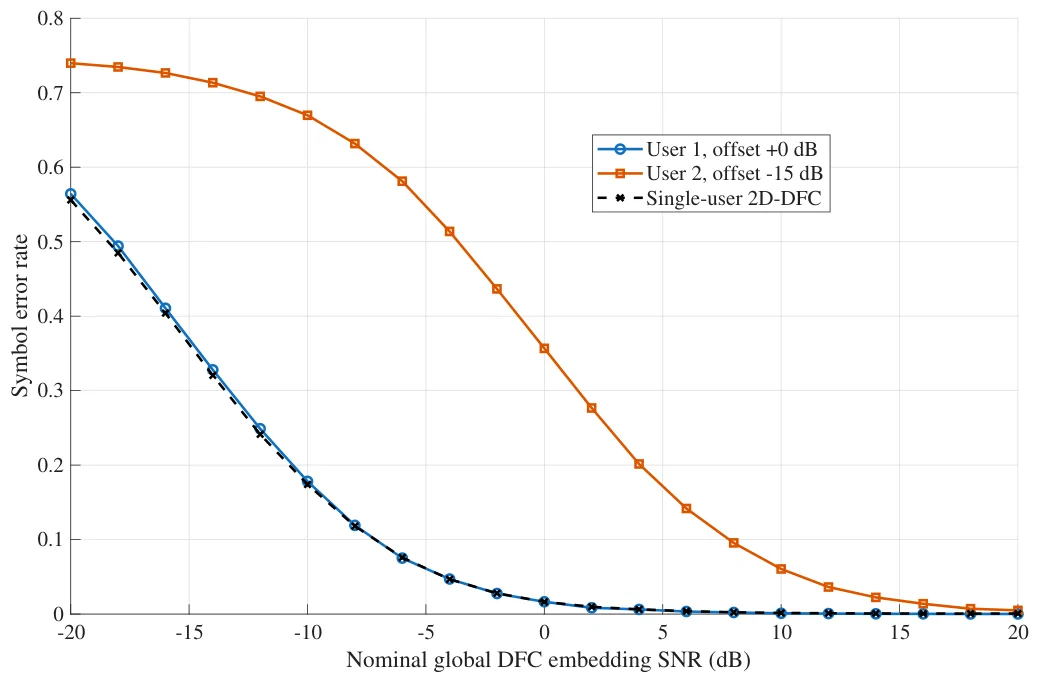
Symbol error rate (SER) performance of the multiuser two-dimensional display field communication (MU-2D-DFC) under the heterogeneous-channel scenario.

Figure 6 displays the SER performance of MU-2D-DFC under the heterogeneous-channel scenario, where User 1 experiences the nominal embedding SNR and User 2 suffers a −15dB SNR offset, modeling one near and one far (or obliquely positioned) viewer.

The SER curve of User 1 closely follows the single-user 2D-DFC baseline over the entire SNR range, confirming that the degraded channel of User 2 does not affect the detection performance of User 1. This result verifies the interference-free property of the proposed orthogonal subband allocation. In contrast, the SER curve of User 2 is shifted to the right by about 15dB. This behavior is expected because User 2 experiences a lower effective SNR at every operating point. For example, when the nominal global DFC embedding SNR is around 0dB, User 1 experiences a low SER, whereas User 2 still experiences a much higher error rate. Therefore, the figure demonstrates the fairness problem caused by equal subband allocation under heterogeneous user conditions.

The parallel shift between the two user curves indicates that the proposed MU-2D-DFC framework does not introduce interuser coupling. The reliability of each user is determined primarily by its own effective SNR. However, this shift demonstrates a severe fairness problem caused by static, equal subband allocation. To mitigate this problem in practical deployments, the MU-2D-DFC framework can be extended to incorporate intelligent, channel-aware resource allocation strategies. Potential approaches include the following:

- Subband reassignment: As demonstrated in (26), the effective SNR highly depends on the localized spectral energy of the input image. A channel-aware scheduler could dynamically assign subbands with stronger spectral energy (i.e., lower-frequency rows with higher $|I_{F}{\left(p,q\right)|}^{2}$) to users experiencing weaker D2C channels to boost their reliability passively.
- Unequal bandwidth allocation: Rather than strict equal partitioning, the system could assign a larger number of frequency rows (a higher $N_{p_{u}}$) to weaker users. This allocation allows users to maintain a target aggregate throughput by transmitting more symbols to compensate for a higher underlying SER.
- Adaptive modulation: The system can adapt the constellation order based on channel conditions, using robust lower-order modulation (e.g., BPSK or 4-QAM) for users with severe SNR degradation while reserving higher-order modulation (e.g., 16- or 64-QAM) for users with strong optical channels.
- Power loading: The transmitter could selectively scale the embedding amplitude of the QAM symbols assigned to the weaker subband of a user. Although this approach increases the effective SNR at the receiver, it must be carefully constrained by a water-filling optimization algorithm to prevent disproportionate visual-quality degradation in the spatial domain.

### 5.4. Per-User Goodput and Sum-Rate Conservation

**Figure 7:**
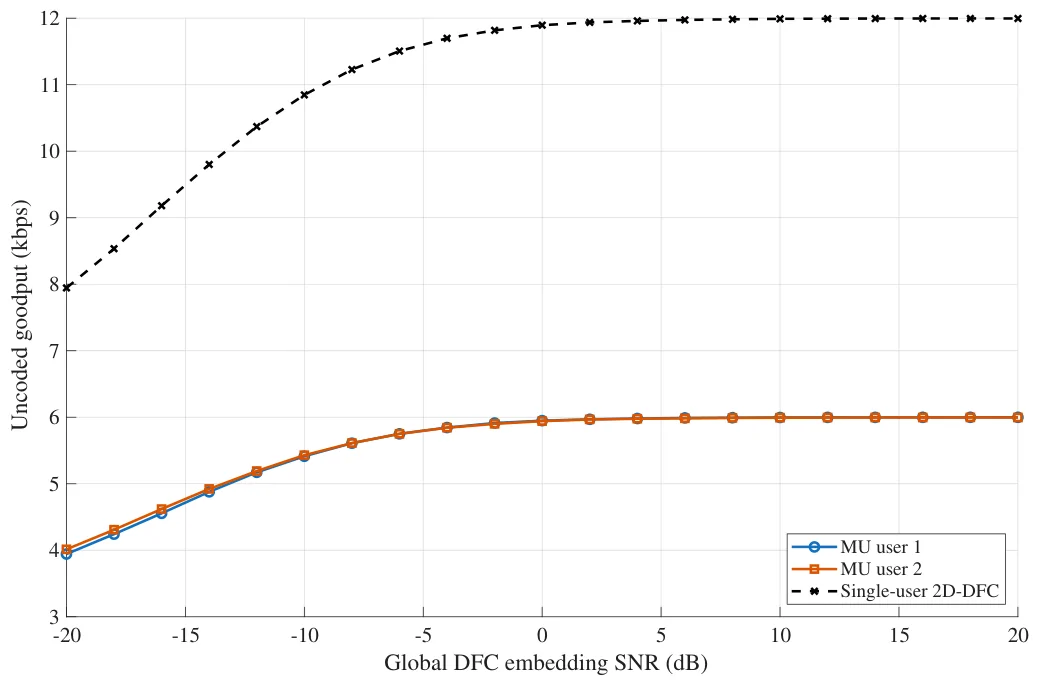
Per-user uncoded goodput of multiuser two-dimensional display field communication (MU-2D-DFC) under the equal-channel scenario.

Figure 7 plots the per-user BER-based goodput for Scenario A alongside the single-user baseline. The goodput for Users 1 and 2 is almost identical over the entire SNR range, which is consistent with the SER behavior in Figure 5. The two users are assigned disjoint subbands with the same number of frequency coefficients; thus, they achieve the same maximum rate. At a high embedding SNR, both users approach their individual raw-rate ceiling of about 6 kbps. In contrast, the single-user 2D-DFC baseline approaches 12 kbps, which is twice the per-user MU rate because the baseline occupies the full 20-row subband, whereas each user occupies only 10 rows. This result confirms that subband partitioning divides the available rate among users without introducing an additional reliability penalty. At a low SNR, the goodput does not drop to zero because the plotted metric is the BER-based uncoded goodput, calculated using $\left(1-BER_{k}\right)$. For random 4-QAM detection, while the SER approaches 0.75, the BER approaches 0.5, causing the uncoded goodput value to tend toward about half of the raw rate. This low SNR value should not be interpreted as practically useful throughput because such frames would require error detection and retransmission in a real system.

**Figure 8:**
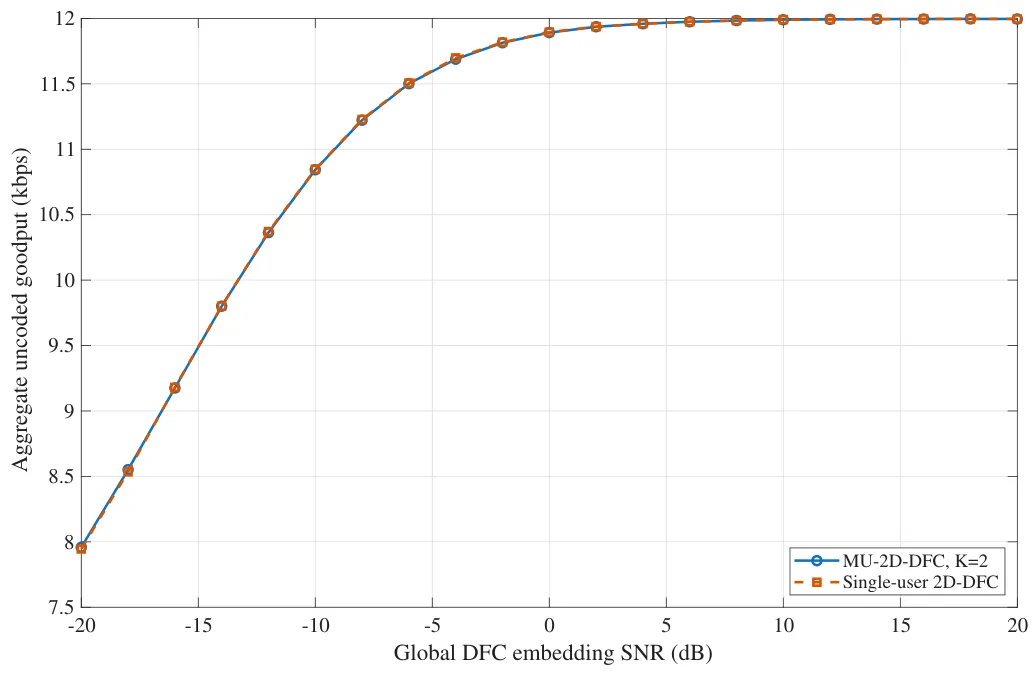
Aggregate uncoded goodput of multiuser two-dimensional display field communication (MU-2D-DFC) with K=2 users compared with the single-user 2D-DFC baseline.

Figure 8 depicts the aggregate uncoded goodput of the proposed MU-2D-DFC scheme with K=2 users and compares it with the single-user 2D-DFC baseline. The two curves overlap across the entire embedding-SNR range, confirming the bandwidth-conservation property of the proposed subband partitioning. Although the total available subband is divided between two users, the aggregate goodput is equal to that of the single-user system occupying the full subband.

At a high embedding SNR, both systems approach the same maximum aggregate goodput of about 12kbps, agreeing with the analytical results of (29) that, under a fixed total bandwidth, the sum rate of MU-2D-DFC is conserved and equals the rate of the corresponding single-user 2D-DFC system. Therefore, the proposed MU transmission does not create an additional sum-rate loss; instead it only redistributes the available throughput among the users. At a low SNR, the aggregate goodput is around 8 kbps rather than decreasing to zero due to the uncoded BER-based goodput definition, where random detection leads to a BER ≈0.5. Thus, this low-SNR goodput reflects the behavior of the adopted uncoded metric, rather than reliable, useful throughput.

In summary, the results establish that (i) MU embedding preserves visual quality (PSNR=45.6dB for K=2); (ii) orthogonal subband partitioning delivers interference-free per-user performance indistinguishable from single-user 2D-DFC at equal-channel quality; (iii) heterogeneous channels translate into per-user SER offsets, motivating adaptive allocation; and (iv) the aggregate throughput of the display is conserved under partitioning. The following additional question is addressed: What is gained by admitting more users when per-user bandwidth is fixed?

### 5.5. Multiplexing Gain and Image-Quality Tradeoff

The results of the previous section addressed the regime with a fixed total bandwidth, in which admitting more users subdivides a constant subband. This section analyzes the complementary regime, where each user is guaranteed a fixed allocation of $N_{p_{u}}=5$ rows, and admitting additional users expands the total occupied subband.

#### 5.5.1. Multiplexing Gain

**Figure 9:**
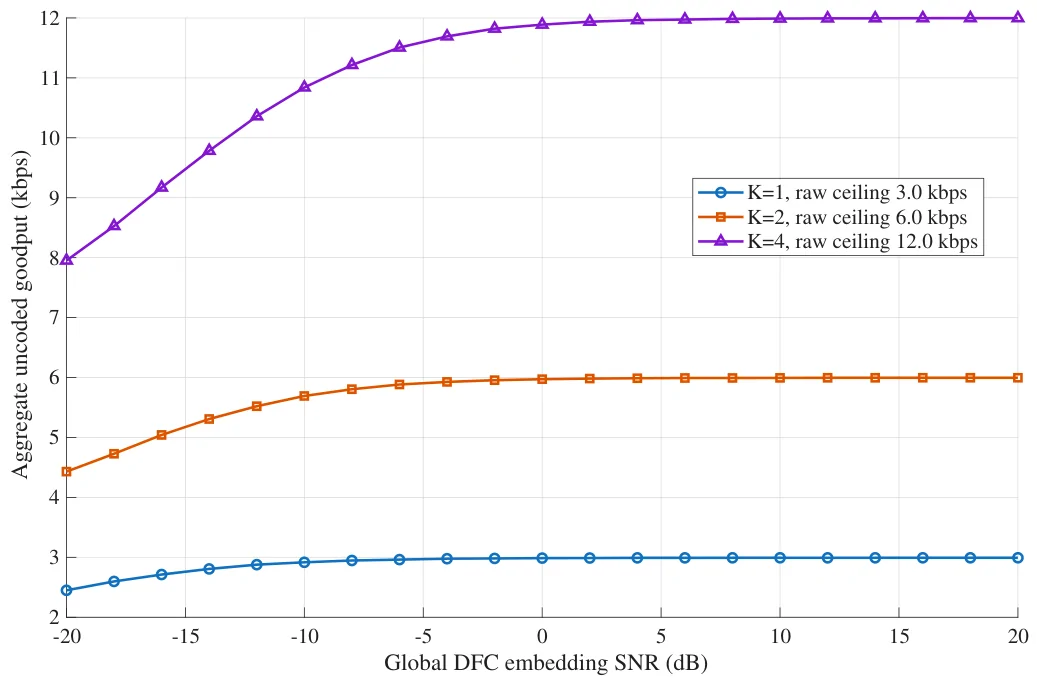
Aggregate uncoded goodput of multiuser two-dimensional display field communication (MU-2D-DFC) for K=1, K=2, and K=4 users under fixed per-user subband (five rows/user) allocation.

Figure 9 presents the aggregate BER-based goodput versus the embedding SNR for K∈{1,2,4} users, each holding a fixed 5×20-coefficient subband. Unlike the case with a fixed total bandwidth, the total occupied subband expands as the number of users increases. Therefore, the aggregate goodput increases almost linearly with *K*. The curves saturate at about 3.0kbps, 6.0kbps, and 12.0kbps for K=1, K=2, and K=4, respectively. These values match the corresponding raw-rate ceilings, confirming that the proposed MU-2D-DFC scheme achieves the ideal multiplexing gain when each user retains a fixed per-user bandwidth.

Linear scaling of the aggregate goodput is achieved in the high-SNR saturation region, where the K=2 and K=4 curves reach 2 × and 4 × the maximum throughput of the K=1 baseline. However, in the transition region (e.g., between −10 dB and 10 dB), the scaling is not strictly proportional at every SNR point. As additional users are admitted, the total spatial-domain perturbation energy increases (corresponding to the 5.1 dB PSNR drop shown in Section 5.5.2). According to the global SNR definition in (31), maintaining the same $\gamma_{global}$ with a larger perturbation power requires a proportionally larger noise variance $\sigma_{k}^{2}$. Consequently, the per-coefficient effective SNR in (26) decreases, causing a slight rightward shift in the transition region of the *K* = 2 and *K*= 4 aggregate goodput curves. Despite this minor shift, the system achieves full multiplexing gain in the operational high-SNR regime, confirming that this scaling is strictly achieved through spatial-frequency bandwidth expansion—activating previously unused 2D DFT subbands—rather than by increasing per-coefficient spectral efficiency. Thus, the demonstrated multiplexing gain refers to the architectural capacity of the system to support additional isolated data streams without concurrently introducing IUI, a capacity bounded by the total available Hermitian-symmetric frequency coefficients rather than by interference limits. 

The transition regions of the three curves maintain a similar profile, demonstrating that the proposed orthogonal subband assignment successfully avoids the severe SNR degradation typically observed in interference-limited multiple-access systems. Therefore, the achievable multiplexing gain is primarily limited by the available Hermitian-symmetric frequency-domain region, rather than by IUI.

The maximum number of users that can be supported in the fixed-per-user-bandwidth regime is limited by the Hermitian symmetry constraint in (4). Each user occupies $N_{p_{u}}$ rows; hence, the total occupied row span for *K* users is $KN_{p_{u}}$. Therefore, the data region must satisfy the following:

(33) $$s_{p}+KN_{p_{u}}<\frac{P}{2}+2.$$

Solving this inequality for *K* results in a maximum admissible number of users of

(34) $$K_{\max}=\left\lfloor \frac{P/2+2-s_{p}-1}{N_{p_{u}}}\right\rfloor .$$

For the simulation parameters $s_{p}=100$, P=256, and $N_{p_{u}}=5$, this equation yields

(35) $$K_{\max}=\left\lfloor \frac{256/2+2-100-1}{5}\right\rfloor =\left\lfloor \frac{29}{5}\right\rfloor =5.$$

Thus, under the present rowwise allocation and single-column-band configuration, at most five users can be assigned nonoverlapping subbands before violating the Hermitian symmetry constraint. This equal rowwise allocation is intended as a simple proof-of-concept to validate the mathematical absence of IUI, rather than an optimal resource allocation strategy.

In practical deployments, the scalability and capacity of MU-2D-DFC can be expanded across several dimensions:

- Image resolution and subband size: Scaling the input image from the evaluated 256×256 resolution to a practical high-definition format (e.g., 1024×1024) increases the total available frequency-domain coefficients by a factor of 16. This massive spectral expansion allows for either a proportional increase in the maximum admissible user count ($K_{max}$) or the assignment of much larger subbands to existing users, significantly increasing per-user throughput without forfeiting visual quality.
- Two-dimensional row-column partitioning: Moving beyond the current 1D rowwise division, partitioning the available frequency region along the row and column dimensions into discrete 2D blocks geometrically increases the combinatorial space for orthogonal user slots. Furthermore, applying the second Hermitian-independent corner pair of the 2D DFT spectrum effectively doubles the available multiplexing region.
- Adaptive QAM and channel-aware allocation: An optimal resource allocation strategy would abandon equal sizing and dynamically adapt to the nonuniform spectral energy of the input image. Users assigned to high-energy (mid-frequency) coefficients could use higher-order modulation (e.g., 16- or 64-QAM) to maximize throughput, whereas users assigned to low-energy (higher-frequency) regions could be allocated physically larger subbands with robust lower-order modulation (e.g., 4-QAM) to maintain reliability.

Developing a full 2D, channel-aware partitioning strategy that dynamically optimizes the subband size and QAM order based on localized image spectral density is a focus for future work.

**Figure 10:**
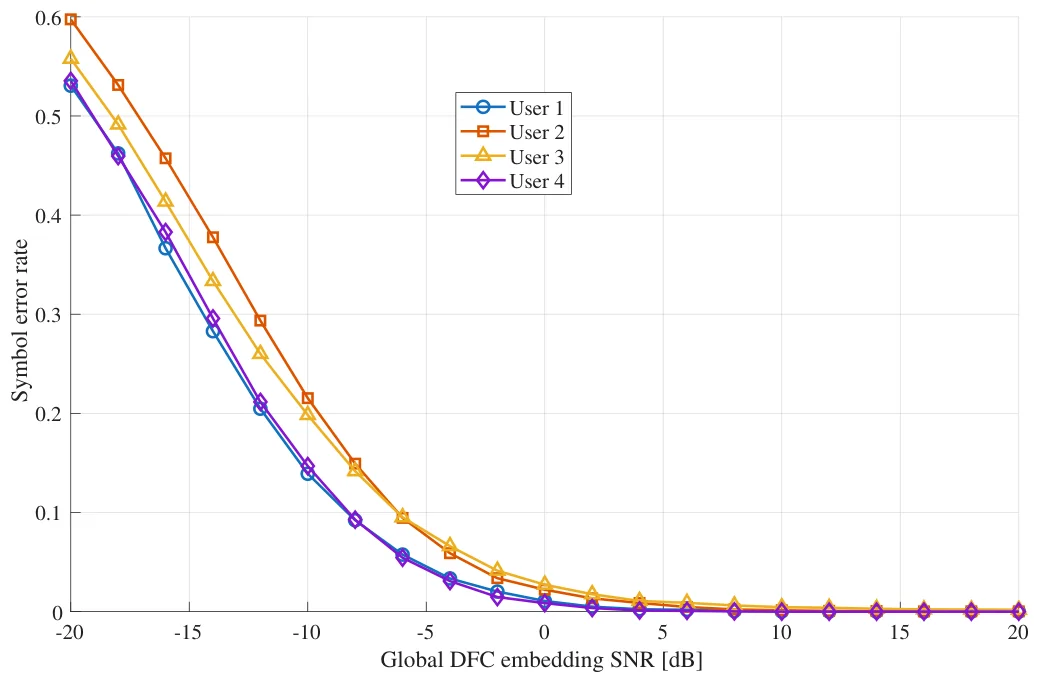
Per-user symbol error rate (SER) of multiuser two-dimensional display field communication (MU-2D-DFC) for K=4 users under a fixed per-user subband allocation.

Although the aggregate goodput scales linearly, evaluating the per-user performance reveals a distinct fairness gap of the natural-image spectrum. Figure 10 plots the individual SER curves for all four users in the scenario with fixed per-user bandwidth and K=4. As analytically predicted by (26), the effective SNR for a user heavily depends on the localized spectral energy, ${|I_{F}\left(p,q\right)|}^{2}$. Rather than exhibiting a strict monotonic decay with increasing row index, the spectral energy distribution in the region near P/2 fluctuates. Consequently, as shown in Figure 10, User 2 acts as the weakest link, while Users 1 and 4 achieve the best error performance. The fairness gap between the strongest and weakest users at an SER of $10^{-1}$ is approximately 2.5 dB. This fairness gap underscores a critical characteristic of MU-2D-DFC: orthogonal frequency partitioning avoids IUI but does not guarantee equal reliability across users due to the non-monotonic, heterogeneous energy distribution of the input image. Achieving strict fairness in future MU-2D-DFC implementations would require adaptive resource allocation strategies, including assigning lower-order QAM constellations to weaker subbands or applying inverse water-filling power allocation to computationally equalize the effective SNR across all active users. 

#### 5.5.2. Analytical PSNR–Multiplexing Tradeoff

**Figure 11:**
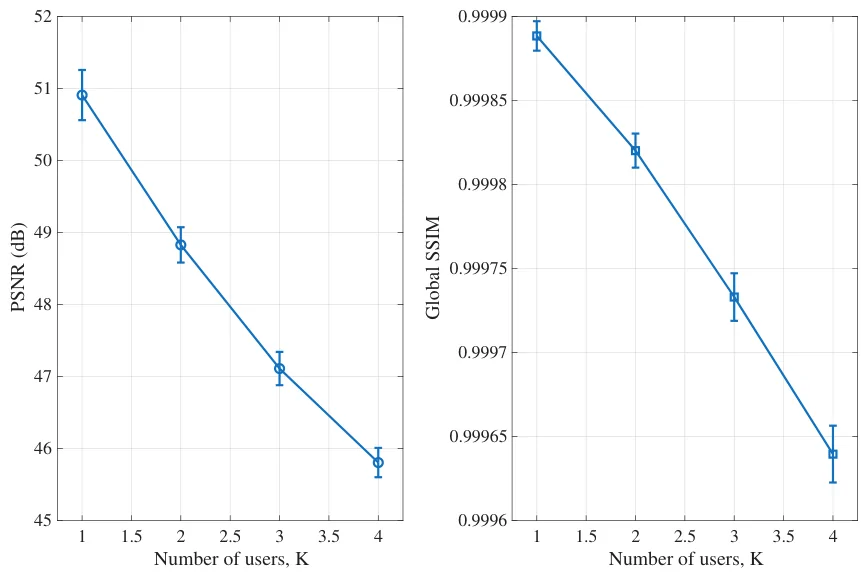
Peak signal-to-noise ratio (PSNR) and structural similarity index measure (SSIM) versus the number of users in the regime with a fixed per-user bandwidth.

Figure 11 illustrates the image-quality cost of increasing the number of concurrently served users in the regime with a fixed per-user bandwidth. The left panel reports the PSNR of the embedded frame, and the right panel reports the corresponding SSIM. As *K* increases from 1 to 4, more frequency-domain coefficients are occupied by user data and their Hermitian mirrors; therefore, the embedding-perturbation energy increases, whereas the visual quality decreases. The measured PSNR decreases from about 50.9dB for K=1 to 45.8dB for K=4. Nevertheless, all PSNR values are far higher than the commonly adopted 30dB perceptual-quality threshold, confirming that the proposed MU-2D-DFC embedding is visually unobtrusive even when four users are concurrently served.

This trend is consistent with the analytical PSNR-multiplexing law. By Parseval’s theorem [28,29], the spatial-domain distortion introduced by the embedding is proportional to the frequency-domain perturbation energy over the occupied subbands and their Hermitian mirrors. Assuming a uniform spectral energy distribution across all allocated frequency rows, the mean-squared error (MSE) would increase linearly with *K*. Under this assumption, the PSNR degradation can be modeled by the following conservative, worst-case upper bound (Appendix C) as follows:

(36) $$\mathrm{PSNR}\left(K\right)\approx \mathrm{PSNR}\left(1\right)-10\log_{10}K.$$

For K=4, this idealized linear model predicts a PSNR reduction of $10\log_{10}4\approx 6.0$ dB. However, the measured reduction is only 5.1 dB. This favorable discrepancy occurs because natural-image spectra are not uniform. As established, the magnitude $|I_{F}\left(p,q\right)|$ tends to decay toward higher-frequency regions. The sequential subbands assigned to later users contain less baseline spectral energy and contribute marginally less spatial-domain perturbation than the initial assumption of linear growth predicts.

The right panel confirms this conclusion from a structural similarity perspective. The SSIM decreases only slightly, from about 0.99989 at K=1 to 0.99964 at K=4. These values are close to unity, indicating that the structural image content is preserved. The tight error bars in both panels reveal that the visual-quality degradation is primarily governed by the subband allocation geometry, that is, how many coefficients are occupied and where they are located, rather than by the random QAM symbols transmitted in each trial. Overall, Figure 11 indicates that the dominant limitation on the number of users is not perceptual image quality but the available Hermitian-symmetric frequency-domain bandwidth. Nevertheless, in the simulated 256×256 frame, all admissible users can be supported while maintaining a large PSNR margin above the perceptual threshold.

Combining these PSNR and SSIM results provides a critical design insight for MU-2D-DFC. Using the conservative approximation in (36) and the measured value PSNR(1)=50.9 dB, the PSNR would theoretically fall below the 30 dB perceptual-quality threshold only when the number of users approaches K≈120. However, this threshold is not a universal constant; instead, it highly depends on the chosen input data and system parameters. If the input data are changed (e.g., by selecting a different input image with a distinct spectral energy distribution), the initial baseline quality (PSNR(1)) would vary, shifting the theoretical point at which the visual quality decreases to below 30 dB. Furthermore, altering the input system parameters dictates the physical multiplexing limit. In (34), the maximum admissible user count imposed by the Hermitian bandwidth constraint is $K_{max}=5$ under the current simulation parameters (256×256 resolution, $s_{p}=100$). Modifying these parameters alters this hard limit. Selecting a smaller starting index $s_{p}$ would increase the admissible number of users (potentially at the cost of the initial visual quality). Using a higher-resolution 1024×1024 frame provides about four times the row budget at the same relative subband placement.

Therefore, for the present configuration, the limiting factor is not the perceptual image quality but the available Hermitian-symmetric spectral region in the 2D DFT domain. In the tested 256×256 image frame, all admissible users can be supported with the full per-user subband allocation while maintaining a visual-quality margin of more than 15 dB over the perceptual threshold. This finding demonstrates that MU-2D-DFC can scale favorably for practical high-resolution signage and display-camera systems, with capacity limits determined dynamically by the input display resolution and input image characteristics.

Alternatively, these results also distinguish the two operating regimes investigated in this study. In the regime with a fixed total bandwidth, admitting more users partitions the constant frequency-domain resource, preserving the aggregate throughput but decreasing the per-user rate. In contrast, in the considered regime with a fixed per-user bandwidth, each admitted user receives the same subband size, so the aggregate throughput increases nearly linearly with *K*. As mentioned, the cost of this multiplexing gain is a logarithmic reduction in the PSNR, which is analytically characterized by (36) and is perceptually insignificant in the admissible spectral range. Thus, MU-2D-DFC provides a flexible design tradeoff between the number of users, per-user rate, aggregate throughput, and image-quality margin.

## 6. Practical Considerations: Geometric, Photometric, and Temporal Distortions

The performance analysis and zero IUI property demonstrated in the preceding sections represent the theoretical upper bounds of the MU-2D-DFC architecture under ideal spatial registration and AWGN conditions. However, in a practical D2C link, the assumption of perfect orthogonality (9) is highly vulnerable to residual geometric distortions.

While standard perspective compensation techniques (e.g., Harris corner detection followed by Hough transform boundary alignment) correct the macro-level off-axis perspective, they are rarely perfect. Residual spatial distortions (including subpixel scaling errors, slight rotational offsets, and resampling interpolation artifacts) cause the spatial-domain pixels to shift relative to the original transmission grid. For instance, simulating an uncompensated rotational offset of just $0.1^{\circ }$ induces severe spectral leakage, degrading the subband signal-to-interference ratio from *∞* to approximately 12 dB, which creates a visible error floor in the high-SNR regime. Furthermore, practical camera hardware introduces lens and motion blurring, which act as spatial low-pass filters. This blurring attenuates the higher-frequency rows of the MU-2D-DFC allocation block more severely than the lower-frequency rows, creating an unequal equalization burden across users. Combined with spectral leakage from imperfect geometric registration, this results in measurable IUI.

Beyond geometric misalignments, practical D2C links are severely affected by photometric and sensor-level impairments. Gamma nonlinearity and sensor saturation (clipping) introduce nonlinear amplitude distortions, which manifest in the frequency domain as intermodulation products and harmonic spectral leakage, degrading subband orthogonality. Furthermore, spatial sampling mismatches between the display pixel grid and the camera sensor grid often generate moiré patterns, overlaying strong, spurious frequency spikes onto the 2D-DFT spectrum. Dynamic illumination changes and ambient-light interference alter the DC bias and low-frequency coefficients, potentially saturating the receiver prior to equalization. Transitioning MU-2D-DFC from this theoretical AWGN framework to experimental real-world deployments would require robust preprocessing frameworks, including strict radiometric calibration (inverse gamma correction) at the receiver, optical anti-aliasing filters to suppress moiré interference, and advanced equalization techniques to track and mitigate these nonlinear DFT-domain distortions dynamically.

In addition to spatial and photometric distortions, a practical MU-2D-DFC implementation must contend with severe temporal and camera-hardware nonidealities. Frame synchronization between the display refresh rate and camera exposure time is critical. Asynchronous capture often results in interframe interference, where a single camera exposure integrates parts of a reference frame and a data-embedded frame. Furthermore, in commodity smartphone cameras, the ubiquitous rolling-shutter mechanism captures images row-by-row rather than instantaneously. Because the 2D-DFT mathematically assumes that the entire spatial frame represents a single point in time, rolling-shutter tearing violates this assumption and scrambles the frequency-domain coefficients, necessitating the use of either global-shutter cameras or highly calibrated exposure-time matching.

Regarding the robustness of the proposed orthogonal subband partitioning under realistic conditions, the architecture exhibits mixed resilience. Because the MU-2D-DFC subbands are positioned in the mid-to-high-frequency regions, the orthogonal partitioning is expected to be highly robust against uniform ambient-light variation, which primarily affects DC bias and extremely low-frequency coefficients. However, the orthogonality is highly vulnerable to nonlinear effects and spatial smearing. Automatic exposure, white balance adjustments, and uncompensated gamma correction introduce nonlinear amplitude shifts that create intermodulation products, causing spectral leakage across the strictly defined user boundaries. Similarly, motion blur functions as a directional low-pass filter that disproportionately attenuates the higher-frequency rows, severely penalizing users assigned to those subbands and exacerbating the fairness gap. While the theoretical orthogonality holds, practical deployments require strict radiometric calibration (e.g., inverse gamma correction), disabled automated camera enhancements, and potentially frequency-domain guard bands to maintain robust interuser isolation.

In real-world deployments, the strict mathematical isolation between $S_{i}$ and $S_{j}$ degrades. A fundamental requirement of displaying the data-embedded frame is converting the real-valued spatial-domain matrix back into 8-bit unsigned integers, which necessitates clipping the pixel values to a strict [0, 255] range. As with PAPR clipping in RF OFDM systems, this nonlinear operation theoretically generates out-of-band spectral leakage, posing a risk to subband orthogonality. However, because MU-2D-DFC is constrained by a strict visual imperceptibility requirement (achieving an observed PSNR ≥ 45.8 dB), the embedding amplitude perturbation is exceptionally small. Table 2 reports the precise percentage of 8-bit clipping as a function of the number of users (*K*), alongside the resulting Leakage-to-Signal Ratio (LSR) and SER degradation measured at a baseline SNR of 10 dB. As shown, the percentage of clipped pixels is statistically negligible, occurring almost exclusively at pixels in the input image that are already saturated at absolute black (0) or absolute white (255). As demonstrated by the empirical SER curves in Section 5, which fully incorporate this 8-bit clipping process, the resulting minor spectral leakage is subsumed by the AWGN noise floor and does not meaningfully degrade multiple-access orthogonality for K≤4.

**Figure 12:**
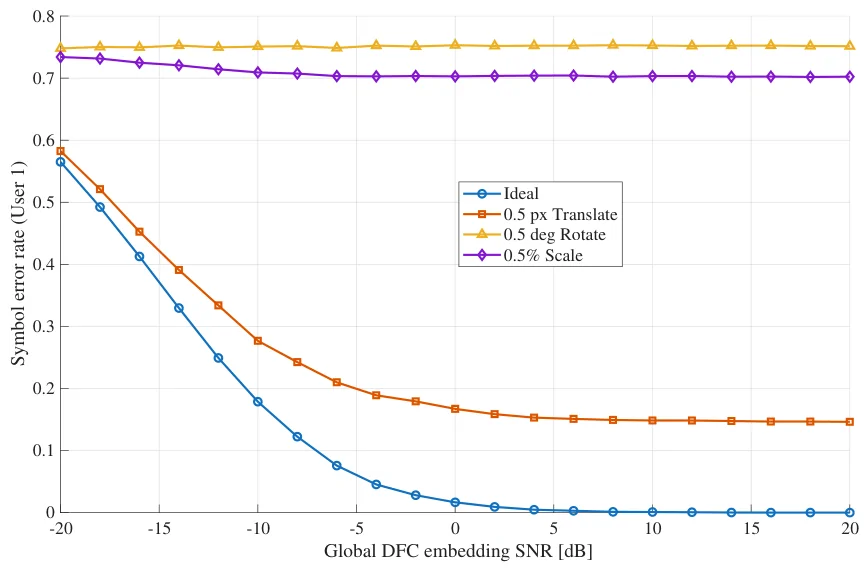
Impact of residual geometric distortions (translation, rotation, and scaling) on the symbol error rate (SER) performance of multiuser two-dimensional display field communication (MU-2D-DFC).

In the 2D-DFT domain, these geometric mismatches act analogously to a carrier frequency offset in RF-OFDM systems. These distortions destroy the strict orthogonality of the frequency bins, causing energy from the designated subband of one user to leak into adjacent subbands. To quantify the vulnerability of the proposed orthogonal partitioning to imperfect spatial registration, Figure 12 plots the SER of User 1 under isolated geometric distortions applied via bilinear resampling prior to equalization. As demonstrated, a sub-pixel translation of 0.5 pixels introduces a severe error floor (SER ≈ 0.15). Because the reference and data frames undergo identical spatial resampling, this degradation is not a simple spatial low-pass effect; rather, it is driven by sub-pixel phase rotation and spectral leakage into neighboring frequency bins caused by the fractional shift near the Nyquist limit. 

**Figure 13:**
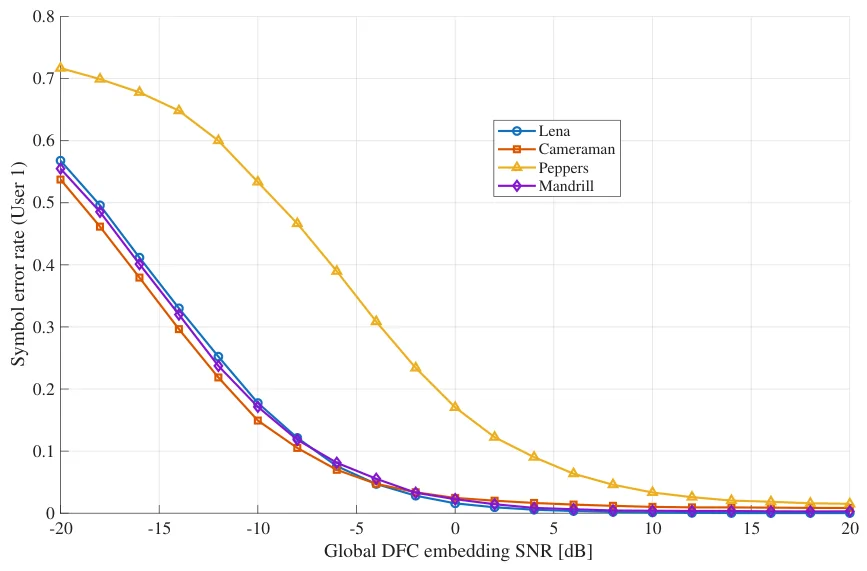
Image dependence of the multiuser two-dimensional display field communication (MU-2D-DFC) framework, illustrating severe symbol error rate (SER) variance across different standard input images.

Conversely, the communication performance is highly dependent on the baseline spectral energy of the displayed content. To quantitatively demonstrate this input-image dependency, Figure 13 plots the SER performance of MU-2D-DFC across four standard test images possessing different spatial-frequency distributions. As predicted by Equation (26), the system’s reliability correlates directly with the localized spectral energy ${|I_{F}\left(p,q\right)|}^{2}$ of the input image in the designated subbands. The ‘Lena’, ‘Cameraman’, and ‘Mandrill’ images possess sufficient localized energy to provide a robust communication channel, achieving near-zero SER at high SNRs. Notably, the ‘Peppers’ image, which consists primarily of smooth, low-frequency gradients, contains less energy in the allocated mid-frequency rows. This causes its effective SNR to drop, shifting its SER curve to the right by approximately 3 to 5 dB relative to the baseline. This powerfully demonstrates that static subband allocation cannot guarantee uniform reliability across all images. To establish comprehensive empirical reliability, future MU-2D-DFC implementations must move beyond single-image baselines and utilize dynamic, channel-aware resource allocation algorithms that assign subbands based on the real-time localized spectral density of the input image. 

**Figure 14:**
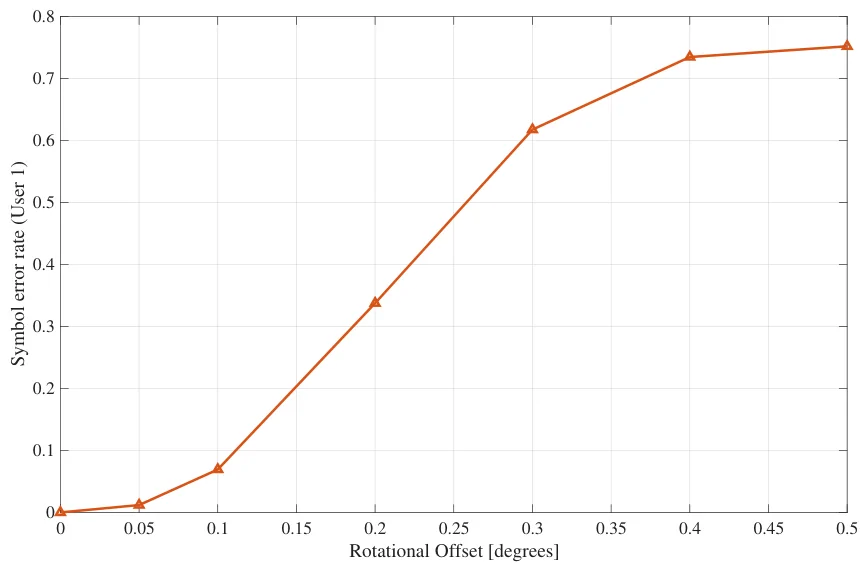
Sensitivity of the multiuser two-dimensional display field communication (MU-2D-DFC) system to uncompensated rotational misalignment.

To explicitly demonstrate this extreme sensitivity to geometric desynchronization, Figure 14 plots the SER of User 1 as a function of continuous rotational offsets (from 0° to 0.5°) at a fixed, high SNR of 20 dB. As shown, even an uncompensated micro-rotation of just 0.1° induces severe spectral leakage and creates a visible error floor. To maintain reliable communication (e.g., keeping $\mathrm{SER}\leq 10^{-2}$ at a 20 dB SNR), the system imposes a strict design requirement: camera-display synchronization must limit rotational misalignment to within $0.05^{\circ }$. As the rotational offset approaches 0.5°, the subband orthogonality is completely destroyed, resulting in total detection failure.

## 7. Conclusions

This study presents MU-2D-DFC, a multiple-access extension of the DFC framework that enables a single electronic display to serve *K* camera-equipped users concurrently through orthogonal 2D frequency subband partitioning. Monte Carlo simulations validated the analytical framework, yielding the following quantitative findings. Under a fixed-total-subband allocation, the aggregate throughput was perfectly conserved. The proposed scheme achieved an aggregate goodput of 12 kbps for K=2 users, matching the single-user 2D-DFC baseline without introducing IUI. Under heterogeneous-channel conditions, a −15 dB SNR imbalance proportionally shifted the affected SER curve of the user, demonstrating that the orthogonal partitioning isolated users and prevented cross-channel error propagation. Under a fixed per-user subband allocation, the aggregate throughput scaled linearly with the number of users, reaching 12.0 kbps for K=4 users. The multiplexing gain incurred a logarithmic cost for the visual quality, governed analytically by $\mathrm{PSNR}\left(K\right)\approx \mathrm{PSNR}\left(1\right)-10\log_{10}K$. Expanding from K=1 to K=4 users reduced the PSNR from 50.9 to 45.8 dB and the SSIM from 0.99989 to 0.99964. These values maintain a substantial margin over the 30 dB perceptual distortion threshold. For the evaluated 256×256 display resolution and selected mid-frequency subband geometry ($s_{p}=100$), the multiplexing limit was strictly bounded at $K_{\max}=5$ users by the Hermitian symmetry constraint, confirming that the system is limited by the available spectral region rather than perceptual degradation.

Although this study establishes the theoretical upper bounds and mathematical framework of MU-2D-DFC in a controlled simulation environment, transitioning this architecture to real-world deployments is a critical next step. Future work should focus on the development of a multicamera-hardware testbed to validate the resilience of the system empirically against the practical geometric, photometric, and synchronization impairments discussed in Section 6. Furthermore, empirical evaluations of practical constraints reveal that MU-2D-DFC performance is highly dependent on both the input-image spatial-frequency spectrum and sub-pixel registration accuracy. While high-texture input images provide robust reliability, smoother images suffer from energy starvation in the data subbands, necessitating the future development of dynamic, channel-aware subband allocation. Additionally, strict geometric synchronization is a fundamental design requirement, as rotational offsets exceeding $0.05^{\circ }$ or sub-pixel translations induce severe phase rotations and spectral leakage that rapidly degrade subband orthogonality.

## 8. Patents

A patent application related to the methods and system presented in this manuscript has been filed.

## Figures and Tables

**Table 1:** Default system parameters.

Parameter	Value
Input image dimensions (P×Q)	256×256
No. of users (K)	2
QAM constellation order (M)	4 (4-QAM)
Total subband rows for all users $\left(N_{\mathrm{total}}\right)$	20
Subband rows per user $\left(N_{p_{u}}\right)$	10
Subband columns per user $\left(L_{q}\right)$	20
Subband row start $\left(s_{p}\right)$	100
Subband column start $\left(s_{q}\right)$	100
Display/camera frame rate $\left(f_{\mathrm{fps}}\right)$	30fps
Effective data frame rate $\left(f_{d}\right)$	15fps

**Table 2:** Impact of 8-bit spatial clipping on multiple-access orthogonality (measured at 10 dB SNR).

Number of Users (*K*)	Clipped Pixels (%)	Leakage-to-Signal Ratio (dB)	SER Degradation
1	0.0009%	N/A	0
2	0.0015%	−65.2	<$10^{-5}$
4	0.0010%	−58.4	<$10^{-4}$

## Data Availability

The original contributions presented in this study are included in the article. Further inquiries can be directed to the corresponding author.

## Appendix A. First-Order Taylor Expansion of (23)

This appendix details the first-order Taylor expansion to obtain an approximation of the high signal-to-noise ratio (SNR) of the zero-forcing (ZF)-equalized symbol. Starting from the elementwise ZF estimate in (23), for $\left(p,q\right)\in S_{k}$,

(A1) $${\hat{X}}^{\left(k\right)}\left(p,q\right)=\frac{I_{F}\left(p,q\right)d_{k}\left(r,c\right)+N_{F}^{\prime (k)}\left(p,q\right)}{I_{F}\left(p,q\right)+N_{F}^{\left(k\right)}\left(p,q\right)},$$

where $d_{k}\left(r,c\right)$ denotes the quadrature amplitude modulation (QAM) symbol of user *k* assigned to the frequency-domain location (p,q). Dividing the numerator and denominator of (A1) by $I_{F}\left(p,q\right)$ produces

(A2) $${\hat{X}}^{\left(k\right)}\left(p,q\right)=\frac{d_{k}\left(r,c\right)+\frac{N_{F}^{\prime (k)}\left(p,q\right)}{I_{F}\left(p,q\right)}}{1+\frac{N_{F}^{\left(k\right)}\left(p,q\right)}{I_{F}\left(p,q\right)}}.$$

Let

(A3) $$\epsilon =\frac{N_{F}^{\left(k\right)}\left(p,q\right)}{I_{F}\left(p,q\right)}.$$

In the high-SNR regime, the magnitude of the input-image frequency coefficient is much higher than the reference-frame noise component, that is,

(A4) $$\left|I_{F}\left(p,q\right)\right|\gg \left|N_{F}^{\left(k\right)}\left(p,q\right)\right|.$$

Therefore, |ϵ|≪1. The Taylor series expansion of ${\left(1+\epsilon \right)}^{-1}$ around ϵ=0 is

(A5) $$\frac{1}{1+\epsilon }=1-\epsilon +\epsilon^{2}-\epsilon^{3}+\cdots .$$

Retaining only the first-order term yields

(A6) $$\frac{1}{1+\epsilon }\approx 1-\epsilon .$$

Substituting (A3) into (A6) results in

(A7) $$\frac{1}{1+\frac{N_{F}^{\left(k\right)}\left(p,q\right)}{I_{F}\left(p,q\right)}}\approx 1-\frac{N_{F}^{\left(k\right)}\left(p,q\right)}{I_{F}\left(p,q\right)}.$$

Using (A7) in (A2) yields

(A8) $${\hat{X}}^{\left(k\right)}\left(p,q\right)\approx \left(d_{k}\left(r,c\right)+\frac{N_{F}^{\prime (k)}\left(p,q\right)}{I_{F}\left(p,q\right)}\right)\left(1-\frac{N_{F}^{\left(k\right)}\left(p,q\right)}{I_{F}\left(p,q\right)}\right).$$

Expanding the product in (A8) results in the following:

(A9) $${\hat{X}}^{\left(k\right)}\left(p,q\right)\approx d_{k}\left(r,c\right)+\frac{N_{F}^{\prime (k)}\left(p,q\right)}{I_{F}\left(p,q\right)}-\frac{d_{k}\left(r,c\right)N_{F}^{\left(k\right)}\left(p,q\right)}{I_{F}\left(p,q\right)}-\frac{N_{F}^{\prime (k)}\left(p,q\right)N_{F}^{\left(k\right)}\left(p,q\right)}{I_{F}^{2}\left(p,q\right)}.$$

The last term in (A9) is a second-order noise term because it contains the product of two noise components. Under the high-SNR assumption, this term is much lower than the first-order noise terms and can be neglected. Hence, the ZF estimate becomes

(A10) $${\hat{X}}^{\left(k\right)}\left(p,q\right)\approx d_{k}\left(r,c\right)+\frac{N_{F}^{\prime (k)}\left(p,q\right)-d_{k}\left(r,c\right)N_{F}^{\left(k\right)}\left(p,q\right)}{I_{F}\left(p,q\right)}.$$

Equation (A10) reveals that the equalized symbol comprises the desired QAM symbol $d_{k}\left(r,c\right)$ and a residual-noise term. This residual term is divided by $I_{F}\left(p,q\right)$; therefore, frequency coefficients with a higher input-image magnitude produce lower effective noise after ZF equalization, whereas weak input-image coefficients amplify noise.

## Appendix B. Derivation of the Residual-Noise Variance (25) and Effective SNR (26)

From the first-order Taylor approximation in (24), the equalized symbol at the frequency-domain position $\left(p,q\right)\in S_{k}$ is written as

(A11) $${\hat{X}}^{\left(k\right)}\left(p,q\right)\approx d_{k}\left(r,c\right)+\zeta_{k}\left(p,q\right),$$

where the residual-noise term after ZF equalization is defined as

(A12) $$\zeta_{k}\left(p,q\right)=\frac{N_{F}^{\prime (k)}\left(p,q\right)-d_{k}\left(r,c\right)N_{F}^{\left(k\right)}\left(p,q\right)}{I_{F}\left(p,q\right)}.$$

For notational compactness, let $I≜I_{F}\left(p,q\right),\;d≜d_{k}\left(r,c\right),\;N≜N_{F}^{\left(k\right)}\left(p,q\right),\;N^{\prime }≜N_{F}^{\prime (k)}\left(p,q\right)$. Then, the residual noise can be written as

(A13) $$\zeta =\frac{N^{\prime }-dN}{I}.$$

The reference-frame noise *N* and data-frame noise $N^{\prime }$ are zero mean and mutually independent. Because each is obtained by summing PQ independent spatial-domain noise samples using the unnormalized two-dimensional (2D) discrete Fourier transform (DFT), their frequency-domain variances are $E\;\left[{\left|N\right|}^{2}\right]=E\;\left[{\left|N^{\prime }\right|}^{2}\right]=PQ\;\sigma_{k}^{2}$. Therefore, the conditional expectation of ${\left|\zeta \right|}^{2}$, given a fixed, known transmitted symbol *d*, is

(A14) $$E\left[{\left|\zeta \right|}^{2}|d\right]=\frac{1}{{\left|I\right|}^{2}}E\left[{\left|N^{\prime }-dN\right|}^{2}|d\right].$$

Expanding the squared magnitude results in

(A15) E|N′−dN|2∣d=E|N′|2+|d|2E|N|2−2Red*EN′N*.

As *N* and $N^{\prime }$ are independent zero-mean noise components, the cross-correlation term vanishes (i.e., $E\left[N^{\prime }N^{*}\right]=0$). Therefore,

(A16) $$E\;\left[{\left|\zeta \right|}^{2}\;|\;d\right]=\frac{PQ\;\sigma_{k}^{2}+{\left|d\right|}^{2}PQ\;\sigma_{k}^{2}}{{\left|I\right|}^{2}}=\frac{PQ\;\sigma_{k}^{2}\left(1+{\left|d\right|}^{2}\right)}{{\left|I\right|}^{2}}.$$

Averaging over the transmitted QAM symbols yields

(A17) $$\sigma_{\mathrm{eff}}^{2}\left(p,q\right)=E\left[{\left|\zeta_{k}\left(p,q\right)\right|}^{2}\right]\approx \frac{PQ\;\sigma_{k}^{2}\left(1+E\left[|d_{k}{|}^{2}\right]\right)}{|I_{F}{\left(p,q\right)|}^{2}}.$$

Using the unit-power normalization in (11) (i.e., $E\left[|d_{k}{|}^{2}\right]=1$), the residual-noise variance becomes

(A18) $$\sigma_{\mathrm{eff}}^{2}\left(p,q\right)\approx \frac{2PQ\;\sigma_{k}^{2}}{|I_{F}{\left(p,q\right)|}^{2}}.$$

The effective per-coefficient SNR is defined as the ratio between the average transmitted symbol power and residual-noise variance after equalization. Hence,

(A19) $$\gamma_{k}\left(p,q\right)=\frac{E\left[|d_{k}{|}^{2}\right]}{\sigma_{\mathrm{eff}}^{2}\left(p,q\right)}.$$

Substituting $E[|d_{k}{|}^{2}]=1$ and the above expression for $\sigma_{\mathrm{eff}}^{2}\left(p,q\right)$, we obtain

(A20) $$\gamma_{k}\left(p,q\right)=\frac{{\left|I_{F}\left(p,q\right)\right|}^{2}}{2PQ\;\sigma_{k}^{2}}.$$

This expression demonstrates that the effective SNR is directly proportional to the input-image spectral energy at the selected DFT coefficient and inversely proportional to the noise variance. Therefore, coefficients with larger ${\left|I_{F}\left(p,q\right)\right|}^{2}$ provide more reliable data recovery after ZF equalization.

## Appendix C. PSNR–Multiplexing Law

This section derives the PSNR-multiplexing design rule in (36). For the unnormalized 2D DFT, Parseval’s theorem [28,29] results in $∥D_{t}-I_{t}{∥}_{F}^{2}=\frac{1}{PQ}{∥D_{F}-I_{F}∥}_{F}^{2}$. Moreover, from (15), the frequency-domain perturbation is confined only to the user-allocated subbands and their Hermitian mirror regions. Hence, the spatial-domain MSE for *K* users is written as

(A21) $$\mathrm{MSE}\left(K\right)=\frac{1}{PQ}\cdot \frac{1}{PQ}\sum_{\left(p,q\right)\;\in \;{⋃}_{k}\left(S_{k}\cup {\bar{S}}_{k}\right)}{\left|I_{F}\left(p,q\right)\right|}^{2}\;E\left[{\left|x-1\right|}^{2}\right].$$

For a zero-mean constellation with unit average power, the perturbation factor satisfies E[|x−1|2]=E[|x|2]−2ReE[x]+1=2. If the average input-spectrum energy per occupied coefficient, denoted by $\bar{|I_{F}{|}^{2}}$, is approximately unchanged over the *K* stacked user bands, then the summation in (A21) contains $2KN_{pu}L_{q}$ coefficients of comparable energy. Therefore, the resulting MSE scales nearly linearly with the number of users, yielding

(A22) $$\mathrm{MSE}\left(K\right)\approx K\cdot \mathrm{MSE}\left(1\right)\;\;⟹\;\;\mathrm{PSNR}\left(K\right)\approx \mathrm{PSNR}\left(1\right)-10\log_{10}K.$$

However, for natural images, $|I_{F}\left(p,q\right)|$ tends to decrease with frequency, and the successively assigned user bands experience slightly lower input-spectrum energy. Hence, each additional user band contributes marginally less distortion than that predicted by the constant $\bar{|I_{F}{|}^{2}}$ approximation. Thus, (A22) provides a conservative upper estimate of the PSNR degradation, consistent with the measured 5.1dB reduction compared with the predicted 6.0dB reduction in Figure 11. The small error floor introduced by clipping it to [0,255] and by the 8-bit quantization further compresses the relative PSNR differences across values of *K*.
